# Synthesis, Photophysical and Electronic Properties of Mono‐, Di‐, and Tri‐Amino‐Substituted Ortho‐Perylenes, and Comparison to the Tetra‐Substituted Derivative

**DOI:** 10.1002/chem.202001475

**Published:** 2020-08-18

**Authors:** Julia Merz, Lena Dietrich, Jörn Nitsch, Ivo Krummenacher, Holger Braunschweig, Michael Moos, David Mims, Christoph Lambert, Todd B. Marder

**Affiliations:** ^1^ Institute for Inorganic Chemistry and Institute for Sustainable Chemistry &, Catalysis with Boron (ICB) Julius-Maximilians-Universität Würzburg Am Hubland 97074 Würzburg Germany; ^2^ Institut für Organische Chemie Julius-Maximilians-Universität Würzburg Am Hubland 97074 Würzburg Germany

**Keywords:** borylation, intersystem crossing, luminescence, polycyclic aromatic hydrocarbon, triarylamine

## Abstract

We synthesized a series of new mono‐, di‐, tri‐ and tetra‐substituted perylene derivatives with strong bis(*para*‐methoxyphenyl)amine (DPA) donors at the uncommon 2,5,8,11‐positions. The properties of our new donor‐substituted perylenes were studied in detail to establish a structure‐property relationship. Interesting trends and unusual properties are observed for this series of new perylene derivatives, such as a decreasing charge transfer (CT) character with increasing number of DPA moieties and individual reversible oxidations for each DPA moiety. Thus, **(DPA)‐Per** possesses one reversible oxidation while **(DPA)_4_‐Per** has four. The mono‐ and di‐substituted derivatives display unusually large Stokes shifts not previously reported for perylenes. Furthermore, transient absorption measurements of the new derivatives reveal an excited state with lifetimes of several hundred microseconds, which sensitizes singlet oxygen with quantum yields of up to 0.83.

## Graphical abstract

**Ortho‐perylenes**: A series of amino‐substituted *ortho*‐perylenes **1**, **2**, and **3**, has been synthesized and their properties compared with those of **4**. The mono‐substituted derivative has the largest CT character and sensitizes singlet oxygen with the highest quantum yield in this series. Transient absorption measurements reveal an additional excited state in each of these perylenes with lifetimes of hundreds of μs.

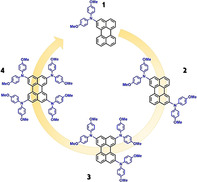

## Introduction

Perylene diimides (PDIs) are one of the most intensively studied classes of functional organic materials due to their thermal, chemical and photochemical stability.^1^, ^2^, ^3^, ^4^, ^5^ Their optical properties are especially interesting as they have quantum yields near unity and exceptional self‐assembly behavior. Hence, PDIs are promising candidates for various applications such as dyes,^6^, ^7^, ^8^ pigments^9^, ^10^ and photonic devices,^5^, ^11^, ^12^, ^13^, ^14^ and the combination of their exceptional optical properties with their electron‐poor character and high charge mobility also makes them promising for organic field effect transistors and organic solar cells.^12^, ^15^, ^16^, ^17^, ^18^, ^19^

**Figure 1:**
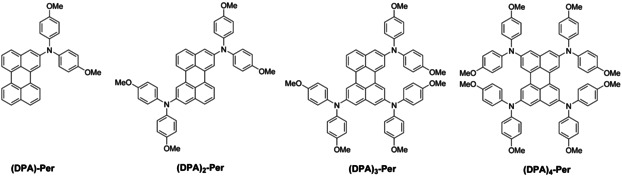
Mono‐, di‐, tri‐ and tetra‐perylenes studied in this paper. We previously reported the tetra‐substituted derivative.^30^

The perylene core possesses 12 positions; however, due to difficulties in functionalizing the core directly, there are relatively few reports on perylene derivatives without carboxyimide groups at the *peri* positions.^20^, ^21^, ^22^ Following our observations^23^, ^24^, ^25^, ^26^, ^27^, ^28^, ^29^ of the interesting electronic and optical properties of 2‐ and 2,7‐substituted pyrene derivatives, due to the ability of π‐donors and acceptors to reorder the frontier orbitals (HOMO−1/HOMO and LUMO+1/LUMO, respectively), we recently reported^30^ tetra‐*ortho*‐substituted perylenes with four bis(*para*‐methoxyphenyl)amine (DPA) or Bmes_2_ (mes=2,4,6‐Me_3_C_6_H_2_) moieties, respectively, at the 2,5,8,11‐positions (*ortho*) which were synthesized via regioselective Ir‐catalyzed C−H borylation.^22^, ^31^, ^32^ These novel derivatives exhibit unprecedented behavior for perylenes, including up to four reversible oxidations or reductions. In addition, strong electronic coupling over the perylene bridge in the mono‐cation radical of **(DPA)_4_‐Per** was observed. The amine‐substituted derivative possesses an unusually long intrinsic lifetime of 94 ns, and transient absorption measurements revealed an additional excited state with a lifetime of 500 μs, which efficiently sensitizes singlet oxygen. Thus, we were motivated to synthesize a series of mono‐, di‐ and tri‐substituted DPA *ortho* perylene compounds to study the impact of the number of DPA moieties on the perylene core at the *ortho* positions both experimentally and theoretically (Figure 1). We were curious to learn whether the above‐mentioned unusual properties could be fine‐tuned by changing the number of DPA units.

## Results and Discussion

### Synthesis

**Scheme 1:**
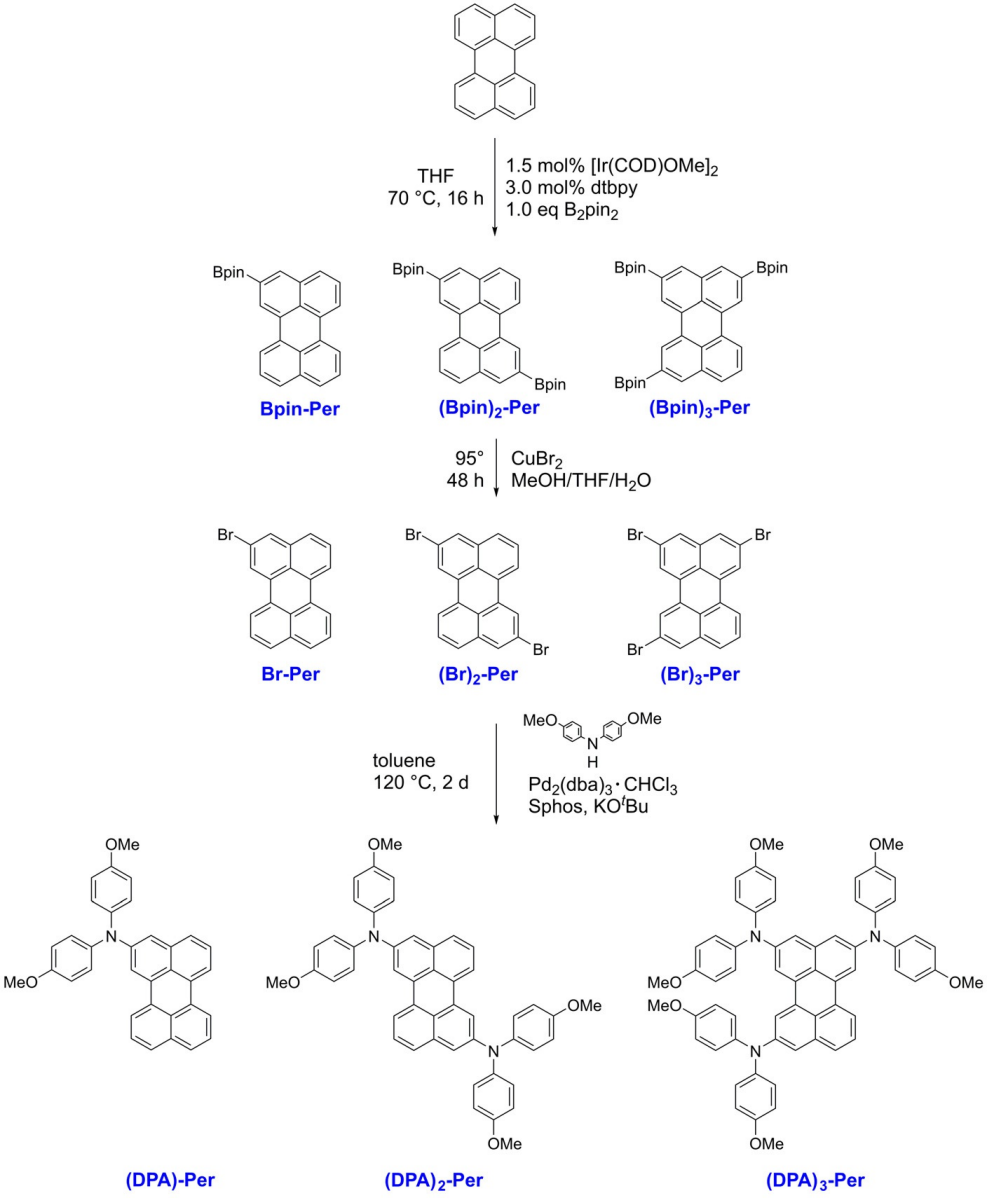
Syntheses of **(Bpin)_1_**_–**4**_ **‐Per**, **(Br)_1_**_–**4**_ **‐Per** and **(DPA)_1_**_–**3**_ **‐Per**. We recently reported the synthesis of **(DPA)_4_‐Per**.^30^

The syntheses of the compounds **(DPA)‐Per**, **(DPA)_2_‐Per** and **(DPA)_3_‐Per** are summarized in Scheme 1. We used the same starting point for the synthesis of the new derivatives as for our previously reported^30^ tetra‐substituted *ortho* perylene **(DPA)_4_‐Per**, which is the high yielding, regioselective Ir‐catalyzed C−H borylation of perylene. In order to obtain the mono‐, di‐, and tri‐borylated derivatives, the number of equivalents of B_2_pin_2_ was reduced from 5 to 1. Thus, a mixture of **Bpin‐Per**, **(Bpin)_2_‐Per**, **(Bpin)_3_‐Per** and some **(Bpin)_4_‐Per** was obtained. The mono‐ and di‐borylated products were successfully separated by column chromatography. Two further regioisomers of **(Bpin)_2_‐Per** are possible, namely 2,5‐bis(Bpin)‐perylene and 2,11‐bis(Bin)‐perylene. However, these two isomers could not be isolated, whereas 2,8‐bis(Bpin)‐perylene (**(Bpin)_2_‐Per**) was isolated successfully. For the follow‐up reactions, the tri‐borylated perylene, in contrast, was used as a mixture containing some residual di‐borylated perylene. THF is the solvent of choice for the borylation reactions, as borylations attempted in MTBE or cyclohexane gave either mostly the higher borylated derivatives and no mono‐ or di‐borylated product or no conversion was observed at all. The borylated compounds **Bpin‐Per**, **(Bpin)_2_‐Per** and **(Bpin)_3_‐Per** were further converted to their corresponding bromo derivatives by halodeboronation,^33^, ^34^ analogously to our previously reported **(Br)_4_‐Per** derivative.^30^ Thus, the respective borylated derivates were suspended in THF and MeOH (1:1) at 50 °C, and CuBr_2_ dissolved in H_2_O was added to the reaction mixture dropwise. The mixtures were stirred at 95 °C for 48 h, until monitoring of the reactions by TLC confirmed full consumption of the starting material. As the brominated derivatives are poorly soluble, only **Br‐Per** was characterized by solution ^1^H NMR spectroscopy, while **(Br)_2_‐Per** and **(Br)_3_‐Per** were used as a mixture for the respective follow‐up reactions. The Buchwald–Hartwig amination reaction was accomplished using Pd_2_(dba)_3_⋅CHCl_3_ as the catalyst precursor and Sphos as the ligand; KO*t*Bu was used as a base and the reaction mixture was stirred at 120 °C for 48 h. The aminated perylene products exhibit good solubility in common organic solvents, and thus purification by column chromatography was possible. All three derivatives **(DPA)‐Per**, **(DPA)_2_‐Per**, and **(DPA)_3_‐Per**, the latter obtained from a mixture of di‐ and tri‐brominated perylene, were isolated in good yields and fully characterized.

### Photophysical properties

**Figure 2:**
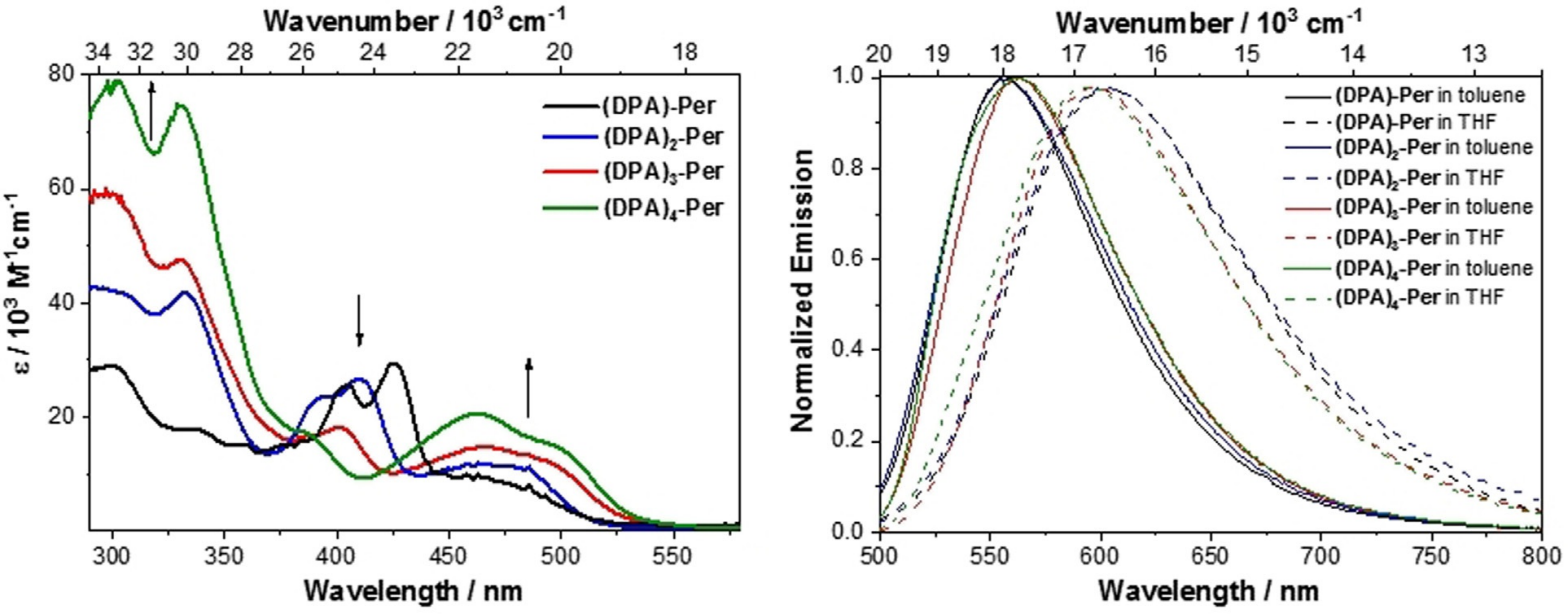
Absorption (left) and emission (right) spectra of **(DPA)‐Per** (black), **(DPA)_2_‐Per** (blue), **(DPA)_3_‐Per** (red) and **(DPA)_4_‐Per** (green) in toluene (solid lines) and THF (dashed lines).

Perylene's S_1_←S_0_ transition has a well‐defined vibronic fine structure with an interval of 1400 cm^−1^ and is allowed with molar absorption coefficients of 34 000 m ^−1^ cm^−1^ at *λ*_max_(abs)=400 nm in toluene. The absorption spectra of our derivatives **(DPA)‐Per**, **(DPA)_2_‐Per**, **(DPA)_3_‐Per** and also, for comparison, our previously reported^30^ **(DPA)_4_‐Per**, are depicted in Figure 2. The lowest energy transition *λ*_max_(abs) of all four compounds is strongly bathochromically shifted in the order **(DPA)‐Per**<**(DPA)_2_‐Per**<**(DPA)_3_‐Per**<**(DPA)_4_‐Per** compared to the parent perylene and is located between 472–499 nm. The intensity of this band follows the same order, with the mono‐aminated derivative having the least allowed S_1_←S_0_ transition with a molar absorption coefficient of 7 000 m ^−1^ cm^−1^ at *λ*_max_(abs)=472 nm while the tetra‐aminated perylene has the most strongly allowed S_1_←S_0_ transition with a molar absorption coefficient of 20 000 m ^−1^ cm^−1^ at *λ*_max_(abs)=499 nm. In contrast to the absorption of perylene, the S_1_←S_0_ transition of the aminated derivatives is rather broad and does not show a vibrational progression. In addition, the absorption spectra indicate that the derivative with the least number of DPA units has the highest charge transfer (CT) character in this series, which decreases with increasing number of donor moieties.

All four derivatives possess rather moderate fluorescence quantum yields (*Φ*=0.15–0.26), emitting in the orange‐red region of the electromagnetic spectrum. Interestingly, the intensity of the fluorescence decreases in the order **(DPA)_4_‐Per**>**(DPA)_3_‐Per**>**(DPA)_2_‐Per**>**(DPA)‐Per**, with the mono‐aminated derivative exhibiting the weakest fluorescence with *Φ*=0.15, while the tetra‐aminated perylene exhibits the strongest fluorescence in this series with *Φ*=0.26 (Table 1). The emission shifts bathochromically with increasing number of DPA units in toluene, which correlates with the expected increase of the HOMO energy with each DPA unit.^35^ Interestingly, **(DPA)‐Per** possesses the strongest solvatochromic effect so that, in THF, the emission shifts bathochromically in the order **(DPA)_4_‐Per**<**(DPA)_3_‐Per**<**(DPA)_2_‐Per**<**(DPA)‐Per**. This implies that the excited state of **(DPA)‐Per** is more polar than that of **(DPA)_4_‐Per**. Hence, the CT (charge transfer) character of these compounds decreases in the order **(DPA)‐Per**>**(DPA)_2_‐Per**>**(DPA)_3_‐Per**>**(DPA)_4_‐Per**, which might be a result of the increased symmetry of the more substituted derivatives.^36^, ^37^ The emission maxima and radiative decay rates of the derivatives **(DPA)‐Per** (*λ*_max_(em)=557 nm, *k_r_*=1.5×10^7^ s^−1^) and **(DPA)_2_‐Per** (*λ*_max_(em)=558 nm, *k_r_*=1.2×10^7^ s^−1^) are very similar to each other, while there are larger differences between **(DPA)_2_‐Per** and **(DPA)_3_‐Per**, and the emission maxima of **(DPA)_3_‐Per** (*λ*_max_(em)=563 nm, *k_r_*=2.0×10^7^ s^−1^) and **(DPA)_4_‐Per** (*λ*_max_(em)=569 nm, *k_r_*=2.2×10^7^ s^−1^) are also very similar to each other. Apparently, the more highly substituted derivatives are stabilized by the polar solvent less efficiently than the two less‐substituted derivatives as they possess less CT character. The apparent Stokes shifts of **(DPA)‐Per** and **(DPA)_2_‐Per**, which are larger than those of **(DPA)_3_‐Per** and **(DPA)_4_‐Per**, also show that the geometry of the former two derivatives undergoes a larger change upon excitation than those of the latter two derivatives, and this is more pronounced in THF than in toluene. Such large Stokes shifts are very unusual for perylene derivatives and have not been reported thus far, to the best of our knowledge.^38^, ^39^ However, this is a necessary property for applications such as imaging and luminescent solar concentrators, as the reabsorption of the emitted light is reduced.^38^, ^39^ Yamato and co‐workers observed a similar trend of the solvatochromism in the series of 1‐(diphenylamino)pyrene, 1,3‐(diphenylamino)pyrene, 1,3,6‐(diphenylamino)pyrene and 1,3,6,8‐(diphenylamino)pyrene.^37^ The intrinsic lifetimes of our four derivatives are quite long compared to that of perylene (*τ*_0_=4 ns), while the tetra‐aminated derivative has the shortest lifetime in this series (*τ*_0_=46 ns), in accordance with the Strickler–Berg relation, as this derivative has the largest molar absorption coefficient.^40^ These are the longest intrinsic lifetimes of perylenes reported to date.

**Table 1:** Selected photophysical data for perylene, **(DPA)‐Per**, **(DPA)_2_‐Per**, **(DPA)_3_‐Per** and **(DPA)_4_‐Per**.^30^

Cpd.	Medium	*λ*_abs_ [nm] (*ϵ* [10^3^ m ^−1^ cm^−1^])	*λ*_em_^[a]^ [nm]	Apparent Stokes shift^[b]^ [cm^−1^]	τ [ns]	Φ	τ_0_^[c]^ [ns]	*k*_nr_^[d]^ [10^7^ s^−1^]	*k*_r_^[e]^ [10^7^ s^−1^]
**perylene^[41]^**	toluene	440 (34)	444	4	4.0	0.95	4.0	1.3	24
**(DPA)‐Per**	toluene	472 (7), 426 (26), 404 (22), 300 (23)	557	3233	10	0.15	66	8.5	1.5
	THF	472, 424, 402, 298	604	4630	7.2	0.07	100	12.8	1.0
**(DPA)_2_‐Per**	toluene	473 (11), 410 (26), 393 (22), 332 (39), 294 (39)	558	3220	19	0.22	86	4.1	1.2
	THF	469, 407, 392, 330, 288	603	4738	8.8	0.07	126	10.6	0.8
**(DPA)_3_‐Per**	toluene	495 (12), 467 (14), 402 (17), 331 (47), 298 (58)	563	2440	12	0.24	51	6.4	2.0
	THF	493, 464, 399, 328, 292	597	3533	8.7	0.10	85	10.3	1.2
**(DPA)_4_‐Per**	toluene	499 (20), 463 (15), 386 (17), 331 (74)	569	2465	12	0.26	46	6.2	2.2
	THF	501,459, 383, 327	593	3096	8.5	0.09	94	10.7	1.1

[a] Excited at the respective λ_abs_ (max) of S_1_←S_0_. [b] The band maxima of the absorption and emission were used to determine the Stokes shift. [c] The intrinsic lifetime was calculated as τ_0_=τ/Φ. [d] The non‐radiative rate constants were calculated as *k*_nr_=(1−Φ)/τ. [e] The radiative rate constants were calculated as *k*_r_=Φ/τ.

### Reactivity with oxygen

**Figure 3:**
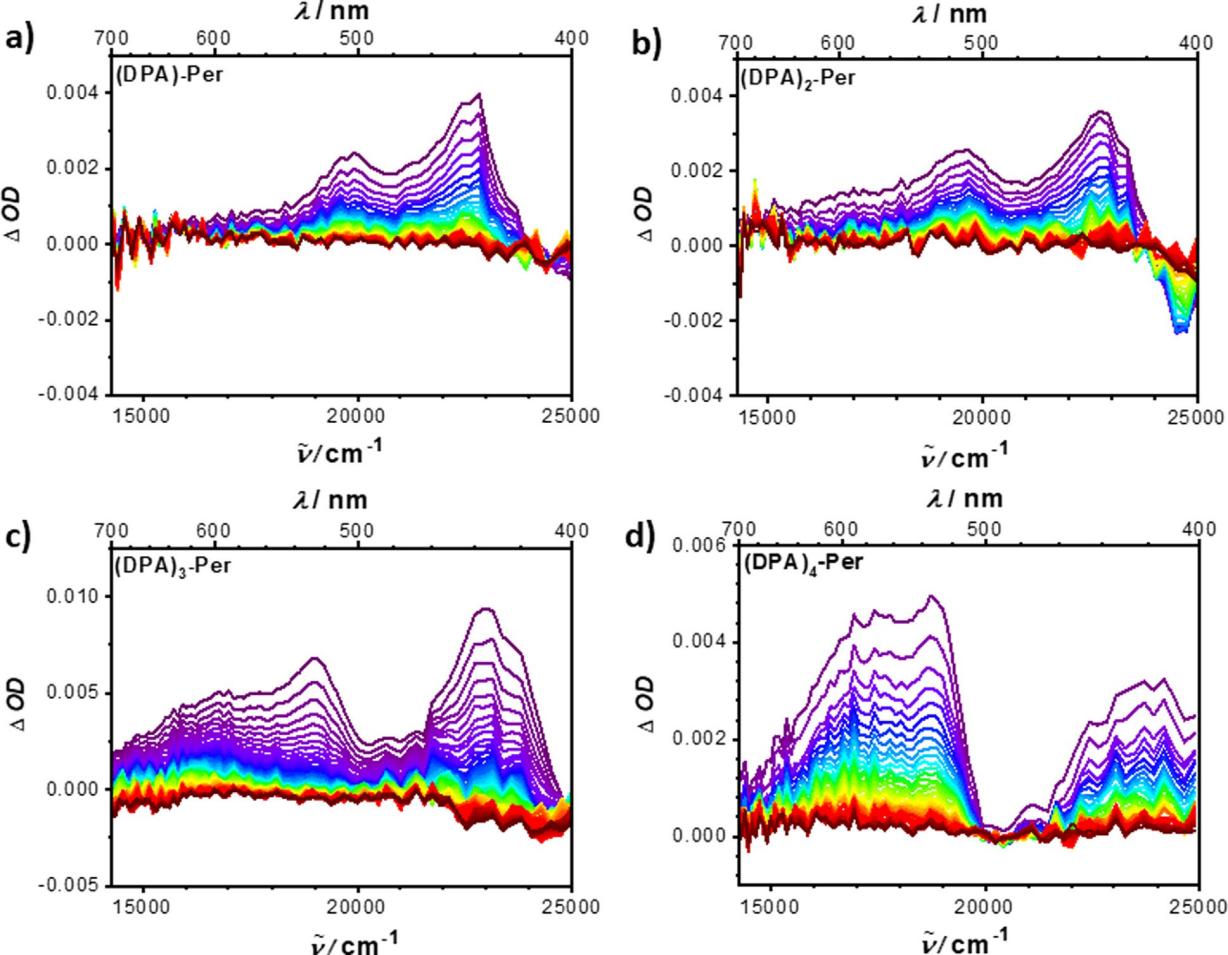
Nanosecond transient absorption spectra in DMF of a) **(DPA)‐Per** as slices every 50 μs after excitation at 23 500 cm^−1^, b) **(DPA)_2_‐Per** as slices every 50 μs after excitation at 23 500 cm^−1^, c) **(DPA)_3_‐Per** as slices every 30 μs after excitation at 21 500 cm^−1^ and d) **(DPA)_4_‐Per** as slices every 50 μs after excitation at 29 400 cm^−1^. Early spectra are depicted in purple to green and later times in red.

In our previous report, we showed that **(DPA)_4_‐Per** can sensitize singlet oxygen with a quantum yield of 0.60, which was detected by its luminescence at 1272 nm upon excitation of an O_2_‐saturated toluene solution.^30^ Transient absorption measurements confirmed the presence of a long‐lived excited triplet state upon photoexcitation, from which energy transfer to the ground state oxygen takes place. Hence, we were motivated to investigate whether the quantum yield of singlet oxygen generation can be fine‐tuned by the number of DPA units. The fluorescence quantum yield Φ_f_ increases with the number of DPA units. Thus, **(DPA)_4_‐Per** exhibits the highest fluorescence quantum yield in this series (Table 2). This indicates that the other derivatives could have an even higher rate of ISC (intersystem crossing) than **(DPA)_4_‐Per** and, consequently, the other derivatives could be more efficient singlet oxygen sensitizers. Compared to the standard, perinaphthenone, for which the quantum yield for generation of singlet oxygen is near unity, **(DPA)_3_‐Per**, **(DPA)_2_‐Per** and **(DPA)‐Per** sensitize singlet oxygen with quantum yields Φ_Δ_ of 0.72, 0.78 and 0.83, respectively (Table 2). To confirm the formation of a possible triplet state upon photoexcitation, we performed transient absorption measurements, which revealed a broad excited state absorption in the range of 400–700 nm with comparable lifetimes (*τ_1_*=150 μs, *τ_2_*=650 μs) to our previously reported **(DPA)_4_‐Per** (Figure 3, Figure S2). Hence, our experiments indeed show that ISC becomes less likely with increasing number of DPA units and, therefore, the quantum yield of singlet oxygen production decreases whereas the fluorescence quantum yield increases.

**Table 2:** Fluorescence quantum yields and quantum yields for singlet oxygen formation.

	Φ_f_	Φ_Δ_
**(DPA)‐Per**	0.15	0.83
**(DPA)_2_‐Per**	0.22	0.78
**(DPA)_3_‐Per**	0.24	0.72
**(DPA)_4_‐Per**	0.26	0.60

### Electrochemistry

**Figure 4:**
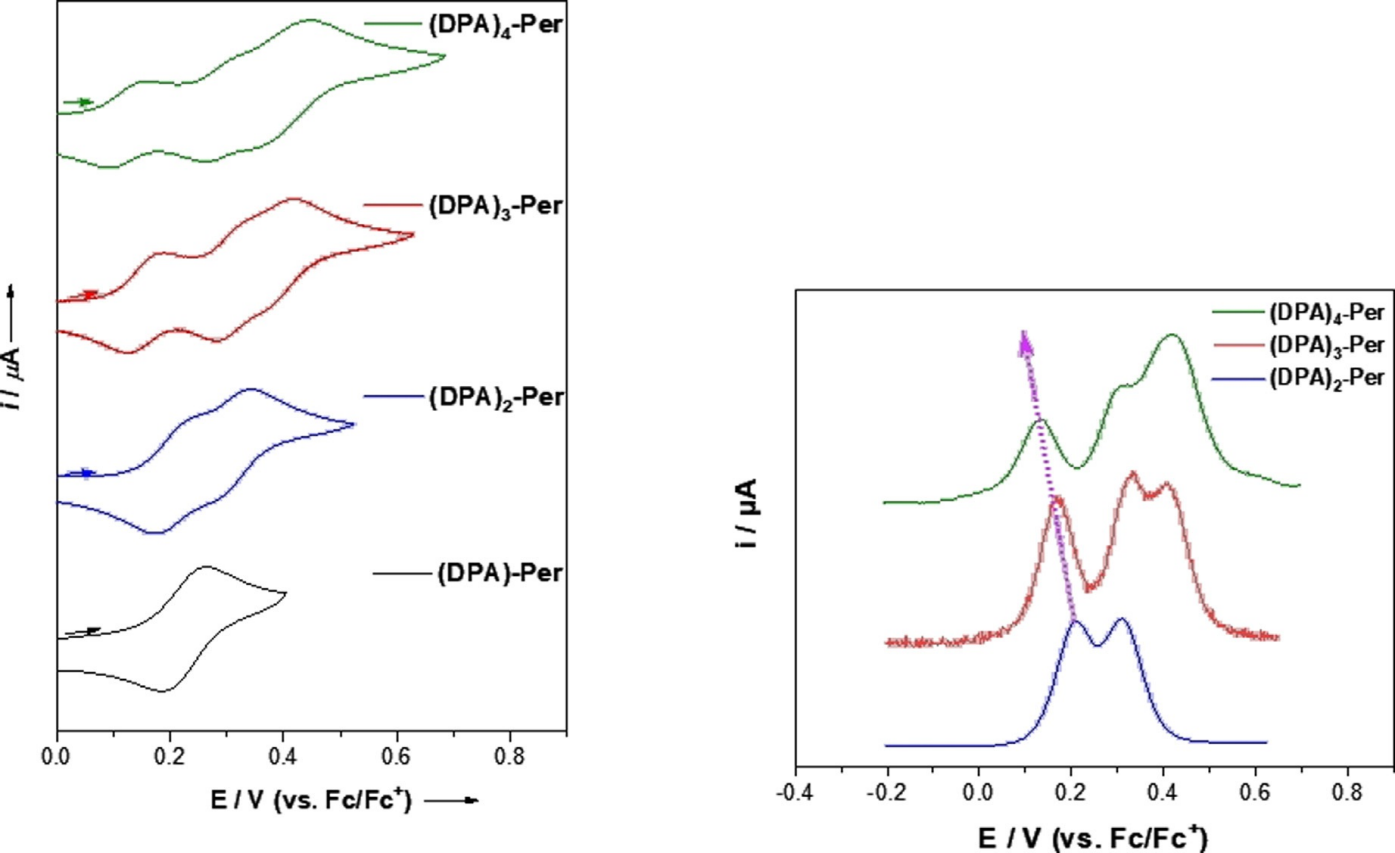
Cyclic voltammograms (left) and corresponding square‐wave voltammograms (right) of (DPA)‐Per (black), (DPA)_2_‐Per (blue), (DPA)_3_‐Per (red) and (DPA)_4_‐Per (green) in CH_2_Cl_2_ with [*n*Bu_4_N][PF_6_] vs. Fc/Fc^+^ with a scan rate of 250 mV s^−1^.

As the tetra‐substituted derivative **(DPA)_4_‐Per** revealed four reversible oxidation potentials, which is unique for perylenes,^30^ we conducted cyclic and square wave voltammetry studies on our new compounds in CH_2_Cl_2_ with 0.1 m [*n*Bu_4_N][PF_6_] and detected one reversible oxidation per DPA unit (Figure 4, Table 3). The first oxidation potential shifts to lower values with increasing number of DPA units. Thus, **(DPA)‐Per** has one reversible oxidation potential at 0.22 V, **(DPA)_2_‐Per** has two reversible oxidation potentials at 0.21 and 0.31 V and **(DPA)_3_‐Per** has three reversible oxidation potentials at 0.17, 0.33 and 0.41 V with respect to Fc/Fc^+^. As previously described,^30^ the third and fourth oxidations of **(DPA)_4_‐Per** are quite close to each other; therefore, we previously conducted a further measurement using the weakly coordinating anion (WCA)‐containing electrolyte [*n*Bu_4_N][Al(OC(CF_3_)_3_)_4_] that is known to separate charged species better in electrochemical studies. Hence, four reversible oxidations at 0.04, 0.24, 0.41 and 0.51 V with respect to Fc/Fc^+^ were detected.^30^ Thus, we now have a series of perylene derivatives which allows one to tune the electrochemical properties depending on the requirements of a particular application.

**Table 3:** Cyclic and square wave voltammetry results from (DPA)‐Per, (DPA)_2_‐Per, (DPA)_3_‐Per, and (DPA)_4_‐Per.^30^ The redox potentials were measured in CH_2_Cl_2_ with [*n*Bu_4_N][PF_6_] as the electrolyte unless otherwise stated. The potentials were referenced to the Fc/Fc^+^ ion couple.

	***E***_**1/2**_	***E***_**1/2**_	***E***_**1/2**_	***E***_**1/2**_
**(DPA)‐Per**	0.22	–	–	–
**(DPA)_2_‐Per**	0.21	0.31	–	–
**(DPA)_3_‐Per**	0.17	0.33	0.41	–
**(DPA)_4_‐Per**	0.13	0.31	0.41	–
**(DPA)_4_‐Per** with WAC^[a]^	0.04	0.24	0.41	0.51

[a] Measured in CH_2_Cl_2_/0.1 m [*n*‐Bu_4_N][Al(OC(CF_3_)_3_)_4_] relative to the Fc/Fc^+^ couple.

### DFT and TD‐DFT calculations

DFT and TD‐DFT calculations were performed to rationalize the observed trends and properties of these perylene derivatives. The ground state structures were first optimized in the gas‐phase at the B3LYP/6‐31G+(d, p) level of theory. Previous studies^23^ have shown that range‐separated hybrid functionals are necessary to obtain a reliable picture of the nature and relative energetic ordering of the excited states. We have thus used the CAM‐B3LYP functional for the subsequent TD‐DFT calculations of the perylene derivatives.

**Figure 5:**
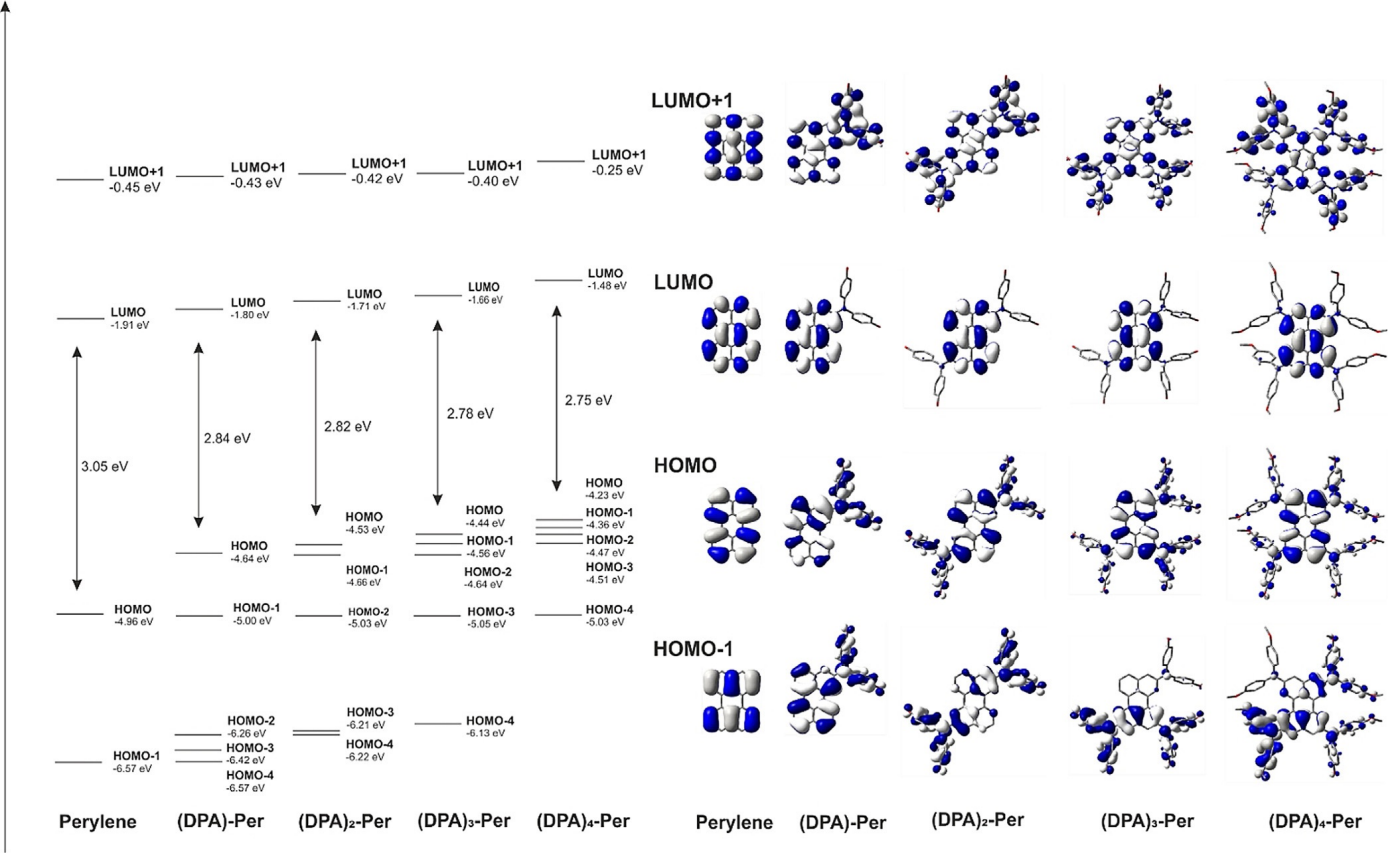
Frontier molecular orbitals (HOMO‐1, HOMO, LUMO, LUMO+1) of perylene, (DPA)‐Per, (DPA)_2_‐Per, (DPA)_3_‐Per and (DPA)_4_‐Per (B3LYP/6–31+G(d, p)). Surface isovalue: ±0.02 [*e* a_0_^−3^]^1/2^.

Unsubstituted perylene has a large HOMO–LUMO gap (3.05 eV) (Figure 5) while the addition of a DPA unit leads to a destabilization of the occupied orbitals as the nitrogen p_z_ orbitals and the π‐orbitals of the methoxy phenyl rings mix very well with the perylene core, and the unoccupied orbitals are less influenced by these substitutions. Therefore, the HOMO–LUMO gap decreases with the addition of DPA units so that **(DPA)_4_‐Per** possesses the smallest gap in the series. However, it is important to note that the destabilization of the HOMO is large upon addition of one DPA unit to perylene, being 0.32 eV, but does not change much with the addition of further DPA units. The lower energy occupied orbitals (HOMO−3 to HOMO−1) are strongly influenced by the DPA units at the *ortho* positions of perylene. Thus, adding one DPA unit destabilizes the HOMO‐1 by 1.57 eV and by 2.21 eV for the tetra‐substituted derivative in comparison to perylene. With the addition of each DPA unit, these lower occupied orbitals are more and more destabilized so that in **(DPA)_2_‐Per**, HOMO and HOMO−1 are almost degenerate, in **(DPA)_3_‐Per**, three orbitals are almost degenerate (HOMO, HOMO−1 and HOMO−2) and, in **(DPA)_4_‐Per**, four orbitals (HOMO to HOMO−3) are nearly degenerate.^30^ Hence, the observed electrochemical behavior is consistent with our calculations, as each DPA unit adds one orbital close to the HOMO of the respective derivative, so that the ease of removing an additional electron from the system increases with the number of substituents.

The TD‐DFT calculations show that the nature of the S_1_←S_0_ transition in all of our derivatives remains HOMO→LUMO in character as in perylene and, as noted previously, for our tetra‐substituted *ortho* perylenes (Table 4).^30^ However, the HOMO→LUMO transition in **(DPA)‐Per** has more CT character, while in **(DPA)_4_‐Per** it has more LE (locally excited) character. Indeed, the proportion of CT character decreases with increasing number of DPA units. This trend is in accordance with the increasing molar absorption coefficients with increasing number of DPA units (**DPA‐Per** *ϵ*=7000 m ^−1^ cm^−1^, **(DPA)_2_‐Per** *ϵ*=11 000 m ^−1^ cm^−1^, **(DPA)_3_‐Per** *ϵ*=12 000 m ^−1^ cm^−1^, **(DPA)_4_‐Per** *ϵ*=20 000 m ^−1^ cm^−1^) and with stronger solvatochromism of the less substituted derivatives, resulting from the CT nature of the lowest excited singlet state. The larger CT character for the lowest energy absorption band for **DPA‐Per** is also reflected in the relatively small oscillator strength (0.300) found for this transition.

**Table 4:** TD‐DFT (CAM‐B3LYP/6–31+G(d, p)) results for the observed major absorption bands of perylene, (DPA)‐Per, (DPA)_2_‐Per, (DPA)_3_‐Per and (DPA)_4_‐Per.

	*S*_n_	*E* [eV] (*E* [nm])	*f*	Assignment
**Perylene**	1	3.21 (387)	0.446	H→L (99 %)
	2	4.06 (305)	0.000	H→L+1 (58 %), H‐1→L (33 %)
	3	4.34 (285)	0.004	H‐2→L (52 %), H→L+3 (38 %)
	4	4.45 (278)	0.000	H→L+2 (42 %), H‐4→L (24 %), H‐1→L (23 %)
	5	4.88 (254)	0.000	H→L+2 (52 %), H‐4→L (30 %), H→L+1 (13 %)
**(DPA)‐Per**	1	3.12 (398)	0.300	H→L (92 %)
	2	3.44 (360)	0.247	H‐1→L (84 %)
	3	4.11 (302)	0.111	H→L+1 (36 %), H→L+2 (20 %)
	4	4.30 (288)	0.146	H‐4→L (21 %), H‐1→L+1 (13 %), H→L+1 (21 %), H→L+3 (19 %)
	5	4.38 (283)	0.028	H‐4→L (10 %), H‐1→L+2 (13 %), H→L+2 (34 %)
**(DPA)_2_‐Per**	1	3.09 (402)	0.320	H→L (93 %)
	2	3.28 (377)	0.002	H‐1→L (85 %)
	3	3.59 (346)	0.386	H‐2→L (81 %)
	4	4.12 (301)	0.000	H→L+1 (36 %), H→L+2 (30 %)
	5	4.33 (86)	0.828	H‐1→L+1 (41 %), H→L+3 (24 %)
**(DPA)_3_‐Per**	1	3.06 (405)	0.320	H→L (92 %)
	2	3.19 (389)	0.100	H‐1→L (84 %)
	3	3.39 (366)	0.133	H‐2→L (83 %)
	4	3.74 (332)	0.232	H‐3→L (77 %)
	5	4.09 (303)	0.107	H→L+1 (46 %), H→L+3 (15 %)
**(DPA)_4_‐Per**	1	3.00 (413)	0.318	H→L (91 %)
	2	3.11 (398)	0.017	H‐1→L (76 %)
	3	3.30 (376)	0.281	H‐2→L (74 %)
	4	3.48 (356)	0.005	H‐3→L (77 %)
	5	3.92 (316)	0.294	H‐4→L (78 %)

## Conclusions

We have synthesized a series of *ortho* perylene derivatives **(DPA)**_***n***_ **‐Per** (*n*=1–3) substituted with one, two and three bis(*para*‐methoxyphenyl)amine (DPA) moieties and compared their properties to those of our previously reported four‐fold substituted species **(DPA)_4_‐Per**. The key step in the synthesis of the new perylene derivatives was the partial Ir‐catalyzed C−H borylation of perylene. We showed that these donor‐substituted perylenes possess tunable photophysical and electrochemical properties. Hence, with increasing number of donor moieties at the *ortho* positions, the CT character of our derivatives decreases while the LE character increases. All of our compounds possess very long intrinsic singlet lifetimes (*τ_0_*=46–126 ns), and transient absorption measurements confirm a further excited state with a lifetime of several hundred microseconds which effectively sensitizes singlet oxygen. Interestingly, the quantum yield for the sensitization of singlet oxygen decreases when more DPA units are added to the *ortho* positions of perylene. Thus, our mono‐substituted derivative sensitizes singlet oxygen with a quantum yield of 0.83 whereas the tetra‐substituted derivative has a Φ_Δ_ value of 0.60. Furthermore, cyclic voltammetry and square wave studies reveal one reversible oxidation per DPA unit, which, to the best of our knowledge has not been observed previously for perylenes. These unique trends are rationalized by theoretical investigations that show that the DPA moieties mix very well with the occupied perylene orbitals, which leads to their strong destabilization. With each DPA unit, the number of occupied orbitals lying energetically close to the HOMO increases, making it easier to remove an increasing number of electrons.

## Experimental Section

General considerations: The catalyst precursors [Ir(COD)(OMe)]_2_ 42 and Pd_2_(dba)_3_⋅CHCl_3_ 43 were prepared according to literature procedures, B_2_pin_2_ was a gift from AllyChem Co. Ltd. while other starting materials were purchased from commercial sources and used as received. Solvents used for synthesis were HPLC grade, further treated to remove trace water using a commercial solvent purification system from Innovative Technology Inc. and deoxygenated using the freeze‐pump‐thaw method.

^1^H, ^13^C{^1^H} and ^11^B{^1^H} NMR spectra were recorded at 298 K using a Bruker Avance 200 (operating at 199.9 MHz for ^1^H, 64.1 MHz for ^11^B{^1^H}, and 188.1 MHz for ^19^F), Bruker Avance 300 III (operating at 300 MHz for ^1^H, 75 MHz for ^13^C{^1^H} and 96 MHz for ^11^B{^1^H}), or Bruker Avance 500 NMR spectrometer (operating at 500 MHz for ^1^H, 125 MHz for ^13^C{^1^H}, 160 MHz for ^11^B{^1^H}, and 470.6 MHz for ^19^F). All chemical shifts (*δ*) were referenced to solvent peaks as follows. ^1^H NMR spectra were referenced via residual proton resonances of CDCl_3_ (^1^H, 7.26 ppm), [D_6_]DMSO (^1^H, 2.50 ppm) and [D_6_]acetone (^1^H, 2.05 ppm). ^13^C spectra were referenced to CDCl_3_ (^13^C, 77.16 ppm), [D_6_]DMSO (^13^C, 39.52 ppm) and [D_6_]acetone (^13^C, 29.84 ppm). ^11^B NMR signals are quoted relative to BF_3_⋅OEt_2_. Chemical shifts are listed in parts per million (ppm) and coupling constants in Hertz (Hz).

HRMS were recorded using a Thermo Scientific Exactive Plus Orbitrap MS system with either an Atmospheric Sample Analysis Probe (ASAP) or by Electrospray Ionization (ESI). Elemental analyses were performed on an Elementar vario MICRO cube elemental analyzer.

Cyclic voltammetry experiments were performed using a Gamry Instruments Reference 600 potentiostat. A standard three‐electrode cell configuration was employed using a platinum disk working electrode, a platinum wire counter electrode, and a silver wire, separated by a *Vycor* tip, serving as the reference electrode. Formal redox potentials are referenced to the ferrocene/ferrocenium ([Cp_2_Fe]^+/0^) redox couple by using decamethylferrocene ([Cp*_2_Fe]; *E*_1/2_=−0.532 V in CH_2_Cl_2_) as an internal standard. Tetra‐*n‐*butylammonium hexafluorophosphate ([*n*Bu_4_N][PF_6_]) or [*n*Bu_4_N][Al(OC(CF_3_)_3_)_4_] were employed as supporting electrolytes. Compensation for resistive losses (*iR* drop) was employed for all measurements.

General photophysical measurements: All photophysical measurements were carried out under an argon atmosphere. All solution state measurements were performed in standard quartz cuvettes (1 cm *x* 1 cm cross section). UV/Vis absorption spectra were recorded using an Agilent 1100 diode array UV/Vis spectrophotometer. Excitation, emission, lifetime and quantum yield measurements were recorded using an Edinburgh Instruments FLSP920 spectrometer equipped with a 450 W Xenon arc lamp, double monochromators for the excitation and emission pathways, and a red‐sensitive photomultiplier (PMT‐R928P) and a near‐IR PMT as detectors. The measurements were made in right‐angle geometry mode and all spectra were fully corrected for the spectral response of the instrument. All solutions used in photophysical measurements had a concentration lower than 10^−5^ M.^.^

Fluorescence quantum yield measurements: Fluorescence quantum yields of the samples were measured using a calibrated integrating sphere (150 mm inner diameter) from Edinburgh Instruments combined with the FLSP920 spectrometer described above. For solution‐state measurements, the longest wavelength absorption maximum of the compound in the respective solvent was chosen for the excitation. In order to exclude self‐absorption, the emission spectra were measured with dilute samples (ca. 0.1 OD at the excitation wavelength).

Fluorescence lifetime measurements: Lifetime measurements were conducted using the time‐correlated single‐photon counting method (TCSPC) on the FLSP920 spectrometer equipped with a high‐speed photomultiplier tube positioned after a single emission monochromator. Measurements were made in right‐angle geometry mode, and the emission was collected through a polarizer set to the magic angle. Solutions were excited with either a 315 (pulse width 932.5 ps), 376 (pulse width 72.6 ps) or a 472 nm (pulse width 90.6 ps) pulsed diode laser at repetition rates of 1–5 MHz and were recorded at emission maxima. Decays were recorded to 10 000 counts in the peak channel with a record length of at least 4 000 channels. The band‐pass of the monochromator was adjusted to give a signal count rate of <20 kHz. Iterative reconvolution of the IRF with one decay function and nonlinear least‐squares analysis were used to analyze the data. The quality of all decay fits was judged to be satisfactory, based on the calculated values of the reduced *χ* ^2^ and Durbin‐Watson parameters and visual inspection of the weighted and autocorrelated residuals.

Transient absorption measurements: Transient absorption spectra were measured with an Edinburgh LP920 laser flash spectrometer equipped with an EKSPLA NT340 Nd:YAG laser with integrated optical parametric oscillator, a 450 W Xe flash lamp, a Hamamatsu R955 photomultiplier and a Tektronix TD3012B oscilloscope for detection of the spectra. The transient maps were obtained by measuring decay profiles in 4 nm steps between ca. 25 000 cm^−1^ (400 nm) and 14 085 cm^−1^ (710 nm). The instrument response (ca. 8 ns) of the set‐up was determined by measuring the scattered light using a LUDOX AS‐30 colloidal silica suspension in water. Decay curves were fitted with the tail‐fit function of the spectrometer software. The quality of all decay fits was judged to be satisfactory, based on the calculated values of the reduced *χ* ^2^ and Durbin–Watson parameters and visual inspection of the weighted and autocorrelated residuals. All solvents were spectroscopic grade and were used without further purification. The sample solutions in the quartz cuvettes were carefully degassed by bubbling argon through the solutions. The samples were excited with ca. 3–6 ns laser pulses at 10 Hz repetition rate. Measurements were performed at pulse energies of 1.2 mJ (excitation at 460 and 550 nm). The stability of the samples was verified by recording the steady‐state absorption spectra before and after the time‐resolved measurements.

Theoretical studies: All DFT and TD‐DFT calculations were carried out with the Gaussian 09 (Rev. E.01) program package^44^ and were performed on a parallel cluster system. GaussView 5.0.9 was used to visualize the results, to measure calculated structural parameters, and to plot orbital surfaces (isovalue: ±0.02 [e a_0_^−3^]^1/2^). The ground‐state geometries were optimized using the B3LYP functional^45^, ^46^, ^47^ in combination with the 6‐31G+(d, p) basis set.^48^, ^49^ The optimized geometries were confirmed to be local minima by performing frequency calculations and obtaining only positive (real) frequencies. Based on these optimized structures, the lowest‐energy gas‐phase vertical transitions were calculated (singlets, 5 states) by TD‐DFT, using the Coulomb‐attenuated functional CAM‐B3LYP^50^ in combination with the 6‐31G+(d, p) basis set.

**(Bpin)‐Per**, **(Bpin)_2_‐Per** and **(Bpin)_3_‐Per**: In an argon‐filled glovebox, a Young's tube was filled with perylene (1.00 g, 3.96 mmol, 1.0 equiv), B_2_pin_2_ (1.0 g, 4.0 mmol, 1.0 equiv), [Ir(OMe)COD]_2_ (39 mg, 60 μmol, 1.5 mol %) and dtbpy (32 mg, 0.1 mmol, 3.0 mol %). The tube was removed from the glovebox, 40 mL of THF was added under argon, and the reaction was stirred at 70 °C for 16.5 h until reaction monitoring by TLC (hexane/CH_2_Cl_2_, 2:1) showed that the starting material was consumed. After removing the solvent, the two products were separated by automated flash column chromatography (Biotage SNAP cartridge KP‐Sil 50 g; 10–15 % CH_2_Cl_2_ in hexane). **(Bpin)‐Per** was obtained as a yellow solid (240 mg, 16 %).^1^H NMR (300 MHz, 298 K, CDCl_3_): *δ*=8.55 (d, 1 H, *J=*1 Hz), 8.33 (dd, 1 H, *J=*1 Hz, *J=*8 Hz), 8.21 (dd, 1 H, *J=*1 Hz, *J=*8 Hz), 8.18 (s, 1 H), 8.16 (dd, 1 H, *J=*0.9 Hz, *J=*8 Hz), 7.72 (d, 1 H, *J=*8 Hz), 7.67 (d, 2 H, *J=*8 Hz), 7.47 (m, 3 H), 1.43 (s, 12 H) ppm; ^13^C{^1^H} NMR (75 MHz, 298 K, CDCl_3_): *δ*=136.0, 134.9, 134.2, 131.4, 131.3, 130.5, 130.4, 129.0, 128.6, 128.0, 127.8, 126.7, 126.6, 126.6, 125.2, 121.3, 120.8, 120.2, 84.2, 25.1 ppm; ^11^B{^1^H} NMR (96 MHz, 298 K, CDCl_3_): *δ*=31.5 (br) ppm; HRMS (APCI): *m*/*z* found: [M]^+^ 379.1864; calc. for [C_26_H_23_BO_2_]^+^ 379.1864 (|Δ|=0 ppm); elem. anal. calc. (%) for C26H23BO_2_: C 82.55, H 6.13; found: C 82.10, H 6.51. **(Bpin)_2_‐Per** was obtained as a yellow solid (361 mg, 9 %). ^1^H NMR (300 MHz, 298 K, CDCl_3_): *δ*=8.53 (d, 2 H, *J=*1 Hz), 8.36 (dd, 2 H, *J=*1 Hz, *J=*8 Hz), 8.18 (s, 2 H), 7.71 (d, 2 H, *J=*8 Hz), 7.47 (t, 2 H, *J=*8 Hz), 1.42 (s, 24 H) ppm; ^13^C{^1^H} NMR (75 MHz, 298 K, CDCl_3_): *δ*=136.1, 134.2, 131.4, 130.5, 130.4, 128.5, 126.6, 125.1, 121.7, 84.2, 25.1 ppm; ^11^B{^1^H} NMR (96 MHz, 298 K, CDCl_3_): *δ*=31.9 (br) ppm; HRMS (APCI): *m*/*z* found: [M]^+^ 505.2721; calc. for [C_32_H_34_B_2_O_4_]^+^ 505.2716 (|Δ|=0.99 ppm); elem. anal. calc. (%) for C32H34B_2_O_4_: C 76.22, H 6.80; found: C 74.85, H 6.88. **(Bpin)_3_‐Per** was obtained with some residual **(Bpin)_2_‐Per** and thus, used as a mixture in the subsequent reactions. HRMS (APCI): *m*/*z* found: [M]^+^ 630.3600; calc. for [C_38_H_45_B_3_O_6_]^+^ 630.3604 (|Δ|=1.11 ppm).

**(Br)‐Per**: To a round bottom flask fitted with a reflux condenser, **(Bpin)‐Per** (260 mg, 0.68 mmol, 1.0 equiv) was dissolved in 10 mL of THF and heated to 50 °C. Then, 10 mL of MeOH was added to this solution and CuBr_2_ (460 mg, 2.1 mmol, 3.0 equiv) dissolved in 10 mL of H_2_O was added and the mixture was stirred at 95 °C for 2 d until reaction monitoring by TLC (hexane/ CH_2_Cl_2_, 2:1) showed that the starting material was consumed. The solution was diluted with water and extracted with CH_2_Cl_2_. The organic phases were collected, and the solvent removed under reduced pressure. The crude product was purified by automated flash chromatography (Biotage SNAP cartridge KP‐Sil 10 g; 10 % CH_2_Cl_2_ in hexane) and obtained as a yellow solid (115 mg, 51 %). The crude solid product was not completely pure due to its low solubility, which did not allow further purification. Therefore, it was not possible to obtain a reasonable ^13^C NMR spectrum or elemental analysis. ^1^H NMR (200 MHz, 298 K, CDCl_3_): *δ*=8.14 (m, 4 H), 7.79 (d, 1 H, ^4^ *J*=2 Hz), 7.72 (d, 1 H, ^3^ *J*=3 Hz), 7.68 (d, 1 H, ^3^ *J*=3 Hz), 7.50 (m, 4 H) ppm; HRMS (APCI): *m*/*z* found: [M]^−^ 332.0041; calc. for [C_20_H_11_Br]^−^ 332.0029 (|Δ|=3.62 ppm).

**(Br)**_***x***_ **‐Per**: To a round bottom flask fitted with a reflux condenser, a mixture of **(Bpin)_2_‐Per** and **(Bpin)_3_‐Per** (730 mg) was dissolved in 35 mL of THF and heated to 50 °C. Then, 35 mL of MeOH was added to this solution and CuBr_2_ (2.31 g) dissolved in 35 mL of H_2_O was added and the mixture was stirred at 95 °C for 2 d until reaction monitoring by TLC (hexane/ CH_2_Cl_2_, 2:1) showed that the starting material was consumed. The solution was diluted with water and extracted with CH_2_Cl_2_. The organic phases were collected, and the solvent was removed under reduced pressure. The crude products of **(Br)_2_‐Per** and **(Br)_3_‐Per** were not completely pure due to their low solubility which did not allow further purification. HRMS (APCI): *m*/*z* found: [M]^−^ 409.9130; calc. for [C_20_H_10_Br_2_]^−^ 409.9134 (|Δ|=0.98 ppm); *m*/*z* found: [M]^−^ 487.8248; calc. for [C_20_H_9_Br_3_]^−^ 487.8239 (|Δ|=1.85 ppm).

**(DPA)‐Per**: In an argon‐filled glovebox, (**Br)‐Per** (140 mg, 0.34 mmol, 1.0 equiv), bis(4‐methoxyphenyl)amine (310 mg, 1.4 mmol, 4 equiv), Pd_2_(dba)_3_⋅CHCl_3_ (9 mg, 8.7×10^−6^ mol, 3 mol %), SPhos (8 mg, 2×10^−5^ mol, 6 mol %) and KO*t*Bu (150 mg, 1.4 mmol, 4 equiv) were added to a Young's tube. The tube was removed from the glovebox, 8 mL toluene was added under argon, and the reaction was stirred at 120 °C for 3 d. The solvent was removed in vacuo and the crude product was purified by automated flash chromatography (Biotage SNAP cartridge KP‐Sil 10 g; 10 % EtOAc in hexane). The desired product was obtained as a red‐orange solid (120 mg, 74 %).^1^H NMR (300 MHz, 298 K, CDCl_3_): *δ*=8.17 (dd, 1 H, *J=*1 Hz, *J=*8 Hz), 8.0 (dd,1 H, *J=*3 Hz, *J=*6 Hz), 7.94 (d, 1 H, *J=*2 Hz), 7.86 (dd, 1 H, *J=*1 Hz, *J=*8 Hz), 7.66 (d, 1 H, *J=*3 Hz), 7.64 (d,1 H, *J=*3 Hz), 7.40 (m, 4 H), 7.14 (m, 4 H), 7.07 (d, 1 H, *J=*2 Hz), 6.88 (m, 4 H), 3.83 (s, 6 H) ppm; ^13^C{^1^H} NMR (75 MHz, 298 K, CDCl_3_): *δ*=156.0, 147.2, 141.1, 136.2, 134.8, 132.2, 131.4, 134.1, 128.9, 128.1, 127.9, 127.1, 126.8, 126.5, 124.7, 120.5, 120.4, 118.3, 117.0, 116.6, 114.9, 55.7 ppm; HRMS (APCI): *m*/*z* found: [M]^+^ 480.1955; calc. for [C_34_H_25_NO_2_]^+^ 480.1958 (|Δ|=0.62 ppm); elem. anal. calc. (%) for C34H25NO_2_: C 85.15, H 5.25, N 2.92; found: C 85.09, H 5.57, N 2.90.

**(DPA)_2_‐Per**: In an argon‐filled glovebox, (**Br)**_***x***_ **‐Per** (277 mg), bis(4‐methoxyphenyl)amine (540 mg, 2.4 mmol), Pd_2_(dba)_3_⋅CHCl_3_ (21 mg, 2.0×10^−5^ mol), SPhos (17 mg, 4.1×10^−5^ mol) and KO*t*Bu (270 mg, 2.4 mmol) were added to a Young's tube. The tube was removed from the glovebox, 20 mL of toluene was added under argon, and the mixture was stirred at 120 °C for 3 d. The solvent was removed in vacuo and the crude product was purified by automated flash chromatography (Biotage SNAP cartridge KP‐Sil 10 g; 10 % EtOAc in hexane). **(DPA)_2_‐Per** was obtained as a red‐orange solid (348 mg, 73 %). ^1^H NMR (300 MHz, 298 K, CDCl_3_): *δ*=7.92 (d, 2 H, *J=*2 Hz), 7.66 (dd, 2 H, *J=*1 Hz, *J=*8 Hz), 7.28 (m, 4 H), 7.14 (m, 8 H), 7.04 (d, 2 H, *J=*2 Hz), 6.86 (m, 8 H), 3.83 (s, 12 H) ppm; ^13^C{^1^H} NMR (75 MHz, 298 K, CDCl_3_): *δ*=156.0, 147.1, 141.1, 136.1, 132.1, 130.8, 126.9, 126.8, 126.6, 124.7, 118.4, 116.8, 116.6, 114.9, 55.6 ppm; HRMS (APCI): *m*/*z* found: [M]^+^ 707.2885 calc. for [C_48_H_38_N_2_O_4_]^+^ 707.2904 (|Δ|=2.68 ppm); elem. anal. calc. (%) for C48H38N_2_O_4_: C 81.56, H 5.42, N 3.96; found: C 81.80, H 5.52, N 4.02.

**(DPA)_3_‐Per**: In an argon‐filled glovebox, **Br_x_‐Per** (484 mg), bis(4‐methoxyphenyl)amine (1.21 g, 5.3 mmol), Pd_2_(dba)_3_⋅CHCl_3_ (37 mg, 3.0×10^−5^ mol), SPhos (29 mg, 7.1×10^−5^ mol) and KO*t*Bu (595 mg, 5.3 mmol) were added to a Young's tube. The tube was removed from the glovebox, 35 mL of toluene was added under argon, and the reaction mixture was stirred at 120 °C for 3 d. The solvent was removed in vacuo and the crude product was purified by automated flash chromatography (Biotage SNAP cartridge KP‐Sil 10 g; 25 % EtOAc in hexane). The product was obtained as a red‐orange solid (348 mg, 26 %). ^1^H NMR (500 MHz, 298 K, [D_6_]acetone): *δ*=7.73 (d, 1 H, *J=*2 Hz), 7.62 (d, 1 H, *J=*8 Hz), 7.55 (d, 1 H, *J=*2 Hz), 7.39 (d, 1 H, *J=*8 Hz), 7.36 (d, 1 H, *J=*2 Hz), 7.27 (t, 1 H, *J=*8 Hz), 7.09 (m, 12 H), 6.97 (d, 1 H, *J=*2 Hz), 6.88 (m, 12 H), 6.66 (d, 1 H, *J=*2 Hz), 6.63 (d, 1 H, *J=*2 Hz), 3.82 (s, 6 H), 3.80 (s, 6 H), 3.79 (s, 6 H) ppm; ^13^C{^1^H} NMR (125 MHz, 298 K, [D_6_]acetone): *δ*=157.4, 157.3, 157.3, 148.7, 148.6, 148.0, 141.6, 141.2, 141.1, 138.3, 137.0, 132.32, 132.30, 132.0, 131.4, 127.9, 127.77, 127.74, 127.6, 124.8, 120.4, 118.8, 116.1, 115.8, 115.64, 115.58, 115.56, 115.0, 114.2, 113.8, 55.71, 55.68, 55.67 ppm; HRMS (APCI): *m*/*z* found: [M]^+^ 934.3822; calc. for [C_62_H_51_N_3_O_6_]^+^ 934.3851 (|Δ|=3.10 ppm); elem. anal. calc. (%) for C62H51N_3_O_6_: C 79.72, H 5.50, N 4.50; found: C 79.57, H 6.42, N 4.10.

## Conflict of interest

The authors declare no conflict of interest.

## Supporting information

As a service to our authors and readers, this journal provides supporting information supplied by the authors. Such materials are peer reviewed and may be re‐organized for online delivery, but are not copy‐edited or typeset. Technical support issues arising from supporting information (other than missing files) should be addressed to the authors.

Supplementary
