# Supplementary material for: Synthesis, Photophysical and Electronic Properties of Mono‐, Di‐, and Tri‐Amino‐Substituted Ortho‐Perylenes, and Comparison to the Tetra‐Substituted Derivative
Source: Chemistry. 2020 Aug 18;26(52):12050–9. doi: 10.1002/chem.202001475 (PMC7540539; doi:10.1002/chem.202001475)
Supplement: Supplementary file 1 — Supplementary [file CHEM-26-12050-s001.pdf]

Chemistry–A European Journal

Supporting Information

Synthesis, Photophysical and Electronic Properties of Mono-, Di-, and Tri-Amino-Substituted Ortho-Perylenes, and Comparison to the Tetra-Substituted Derivative

Julia Merz,[a] Lena Dietrich,[a] Jörn Nitsch,[a] Ivo Krummenacher,[a] Holger Braunschweig,[a] Michael Moos,[b] David Mims,[b] Christoph Lambert,[b] and Todd B. Marder*[a]

Contents

Emission spectra of singlet oxygen sensitization S2

Transient absorption decays S2

NMR spectra S3

Cartesian coordinates S12

Singlet Oxygen Detection

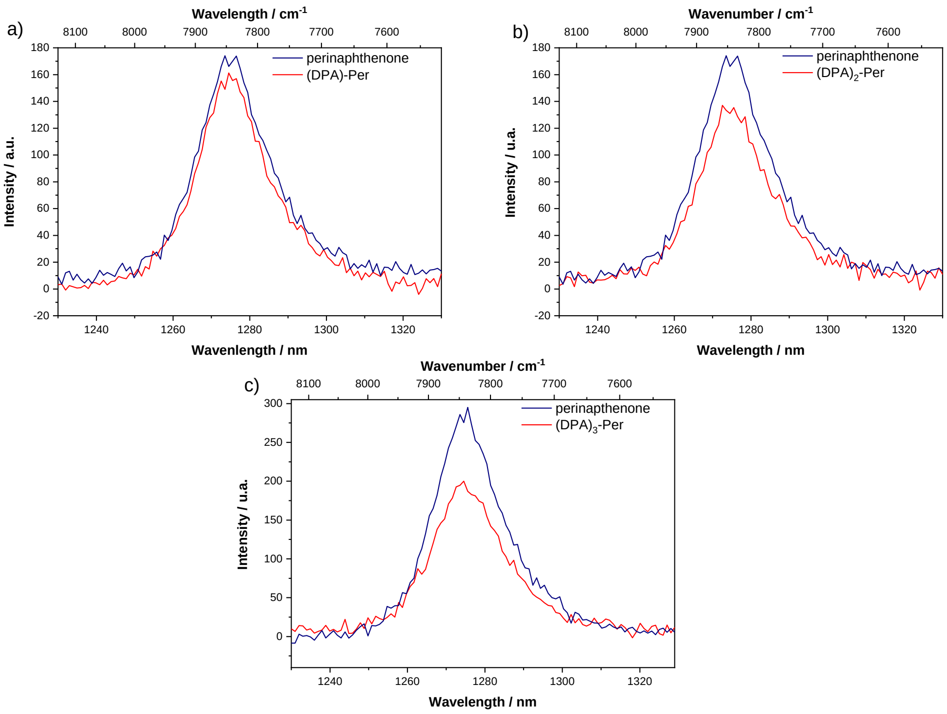

Figure S1: Emission spectra of singlet oxygen generated from sensitization by perinaphthenone (blue) vs. emission spectra of singlet oxygen generated from sensitization by (red) (a) **(DPA)-Per**, (b) **(DPA)$_2$-Per** excited at 410 nm and (c) **(DPA)$_3$-Per** excited at 345 nm.

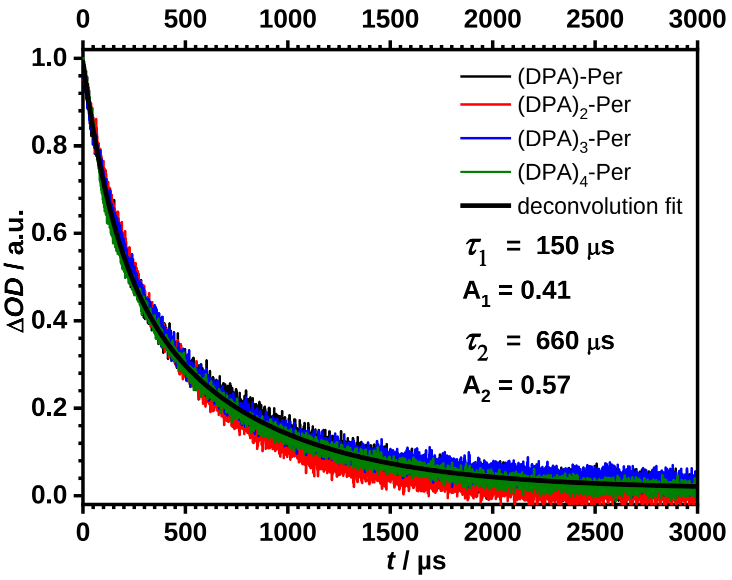

Figure S2: Transient absorption decays of **(DPA)-Per**, **(DPA)$_2$-Per**, **(DPA)$_3$-Per** and **(DPA)$_4$-Per** at the respective wavelength of their maximum (transient) absorption. As these derivatives are not stable, have small concentration variations and the measurements possess rather high S/N rations, all four decays were fitted via Glotaran (v1.2), which results in a lifetime of τ_1 = 150 µs and τ_2 = 660 µs.

NMR Spectra

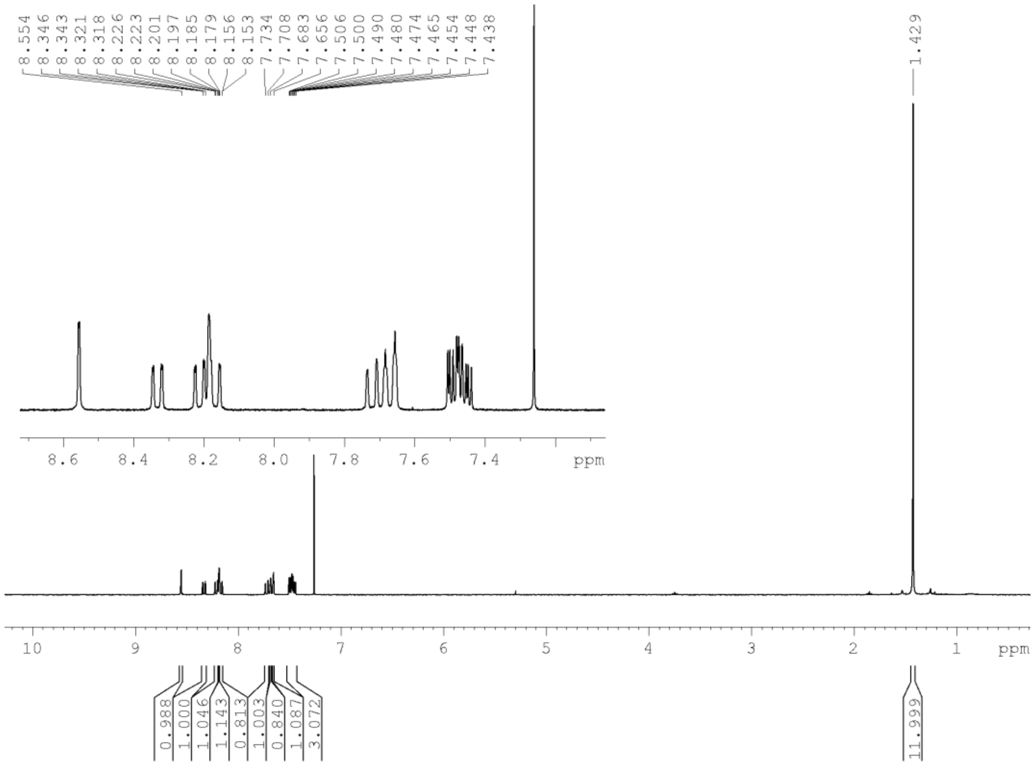

Figure S3: ^{1}H NMR (300 MHz, 298 K) spectrum of **(Bpin)-Per** recorded in $CDCl_3$.

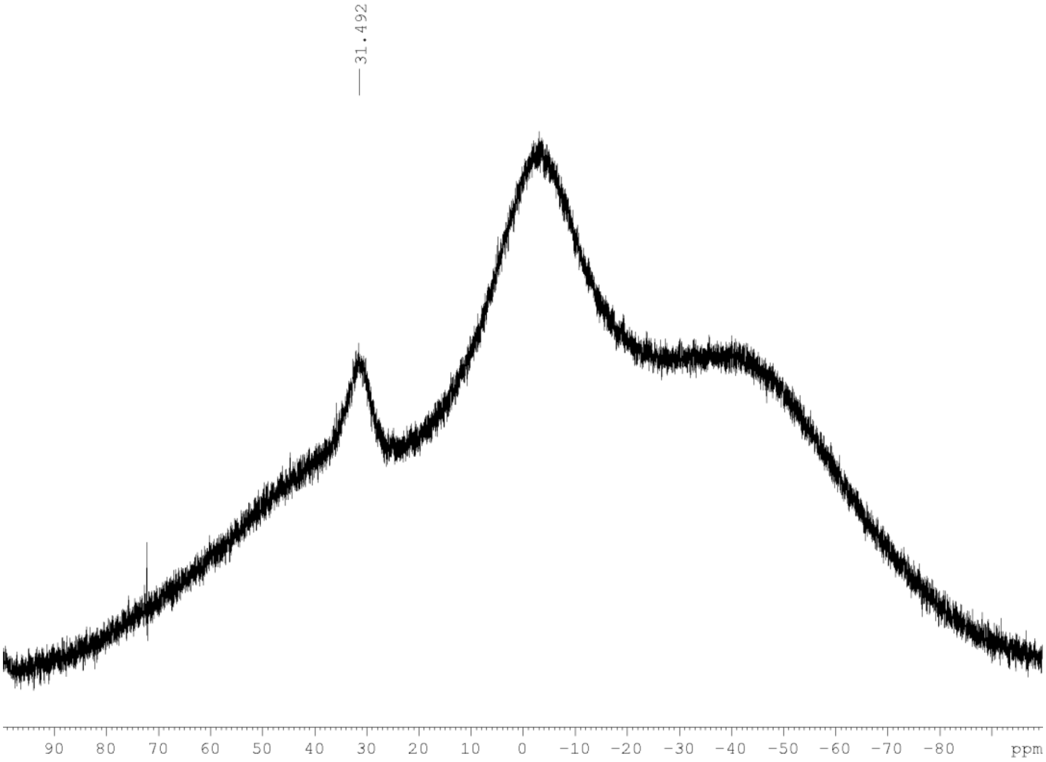

Figure S4: $^{11}B\{^{1}H\}$ NMR (96 MHz, 298 K) spectrum of **(Bpin)-Per** recorded in $CDCl_3$.

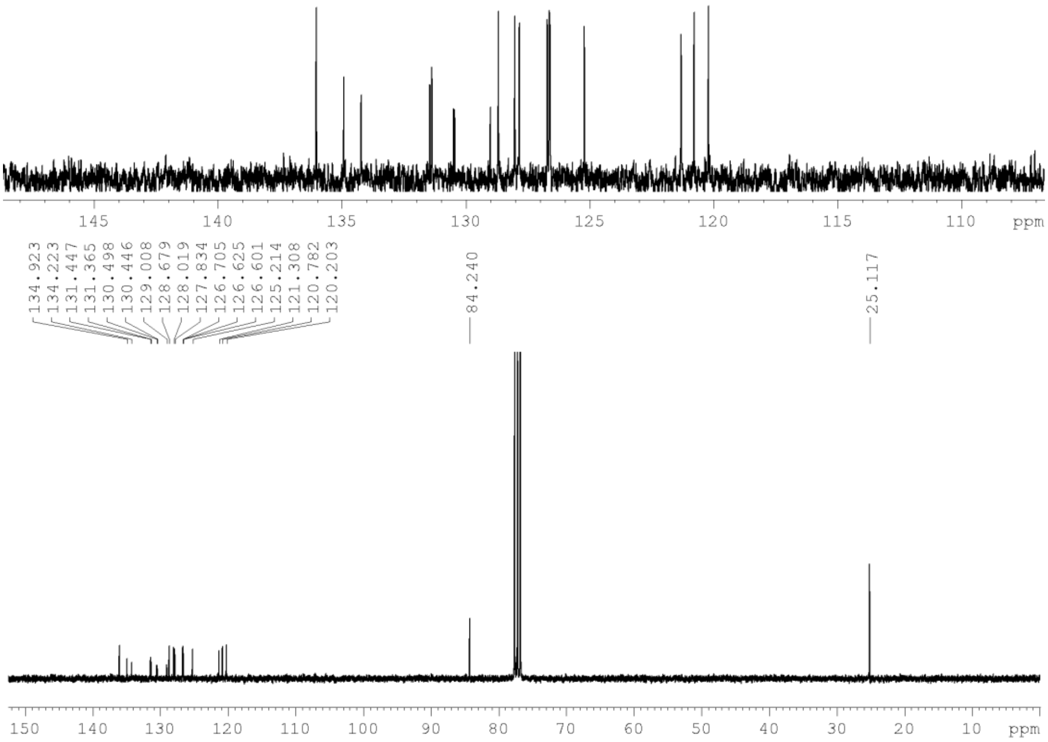

Figure S5: $^{13}C\{^{1}H\}$ NMR (75 MHz, 298 K) spectrum of **(Bpin)-Per** recorded in $CDCl_3$.

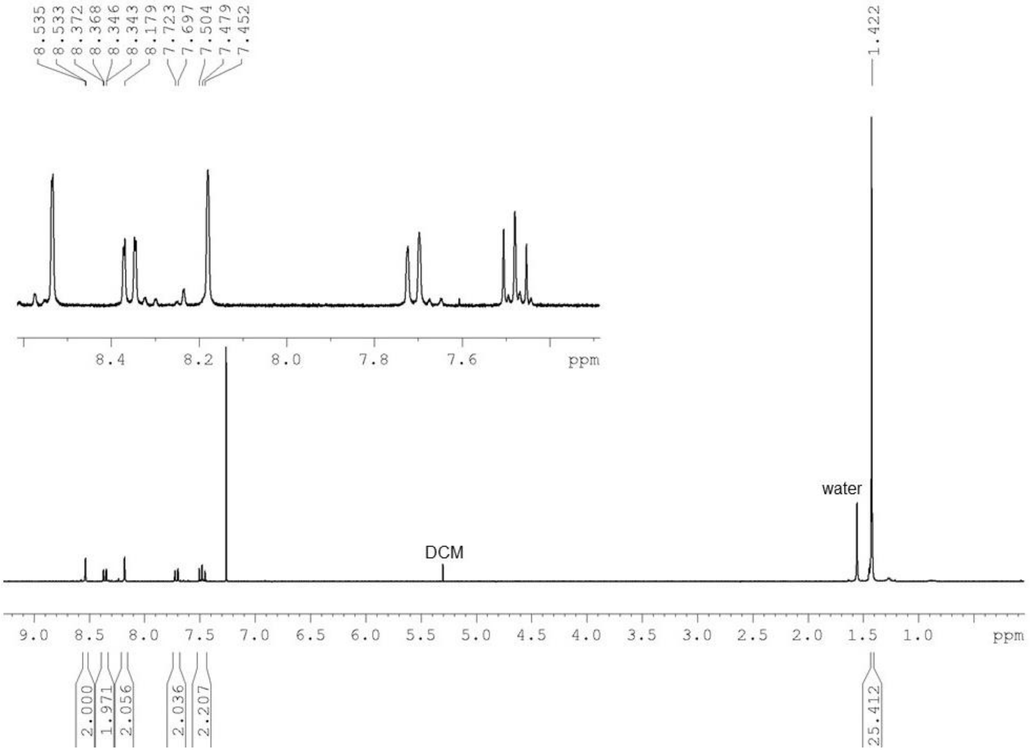

Figure S6: ^{1}H NMR (300 MHz, 298 K) spectrum of **(Bpin)$_2$-Per** recorded in $CDCl_3$.

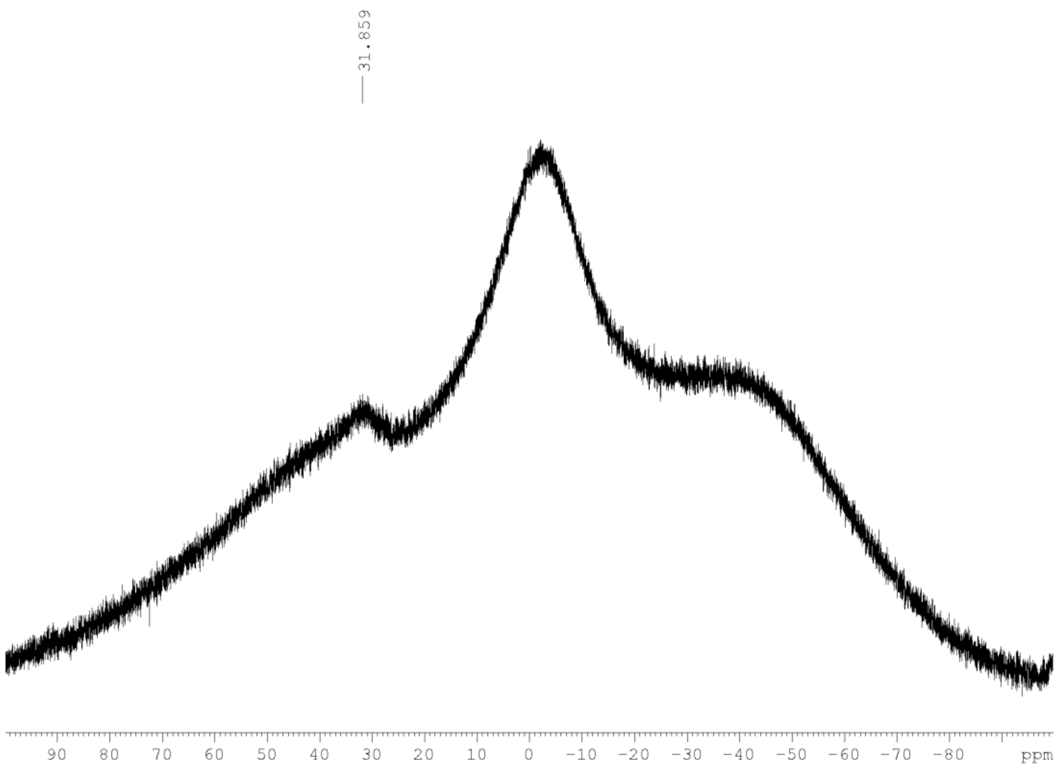

Figure S7: $^{1}B\{^{1}H\}$ NMR (96 MHz, 298 K) spectrum of **(Bpin)$_2$-Per** recorded in $CDCl_3$.

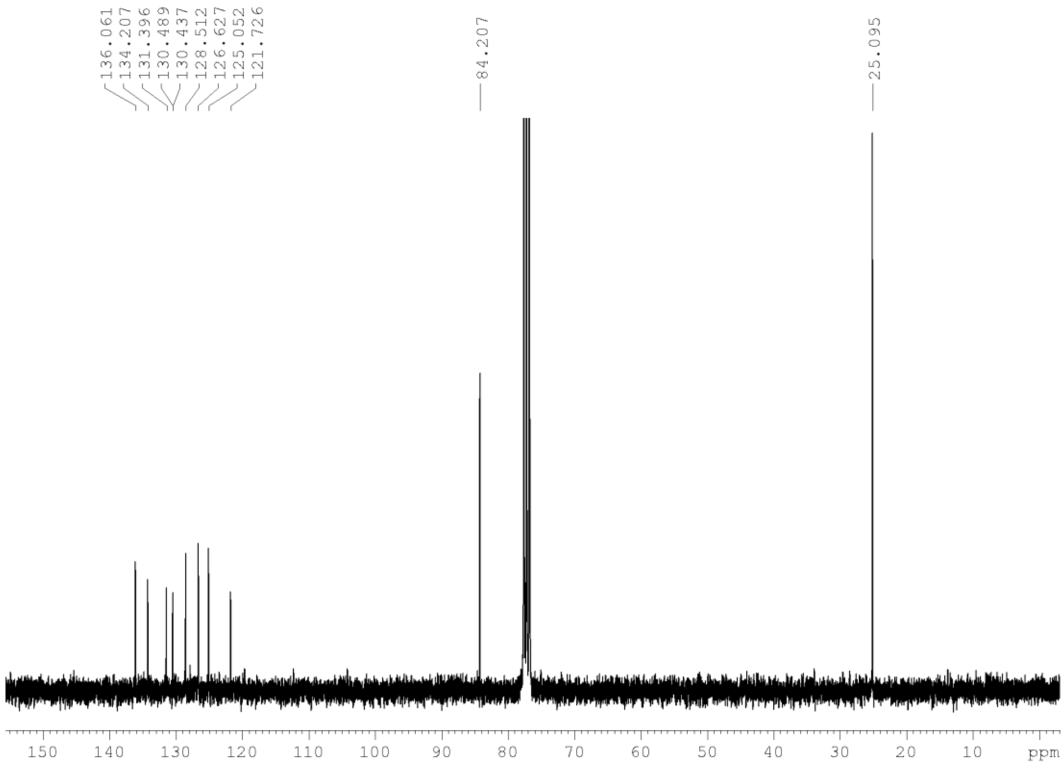

Figure S8: $^{13}C\{^{1}H\}$ NMR (75 MHz, 298 K) spectrum of **(Bpin)$_2$-Per** recorded in $CDCl_3$.

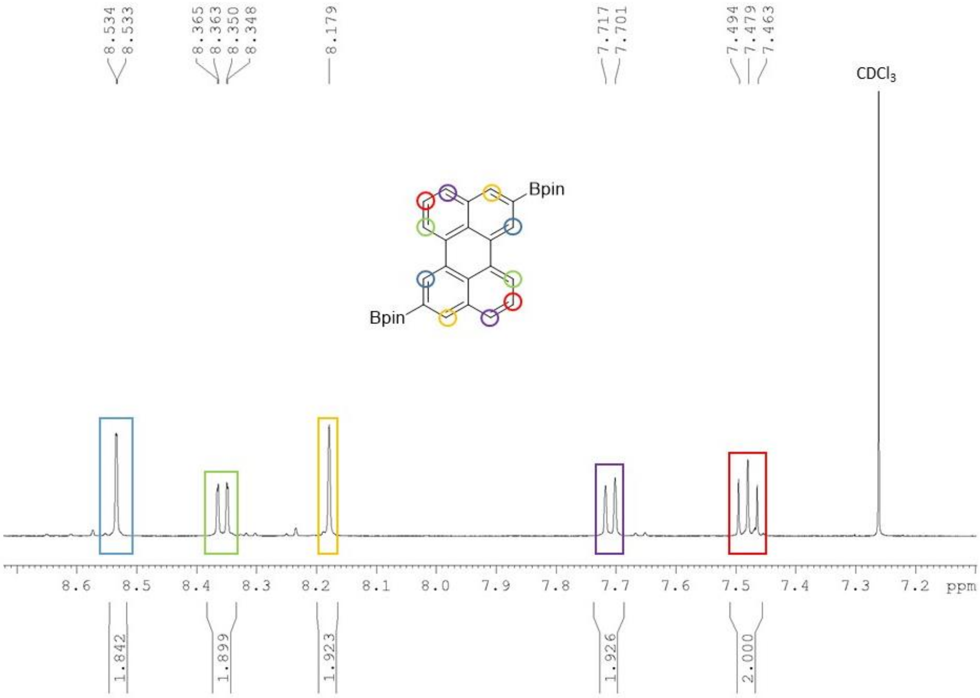

Figure S9: ^{1}H-NMR (500 MHz, 298 K) spectrum **(Bpin)$_2$-Per** recorded in $CDCl_3$.

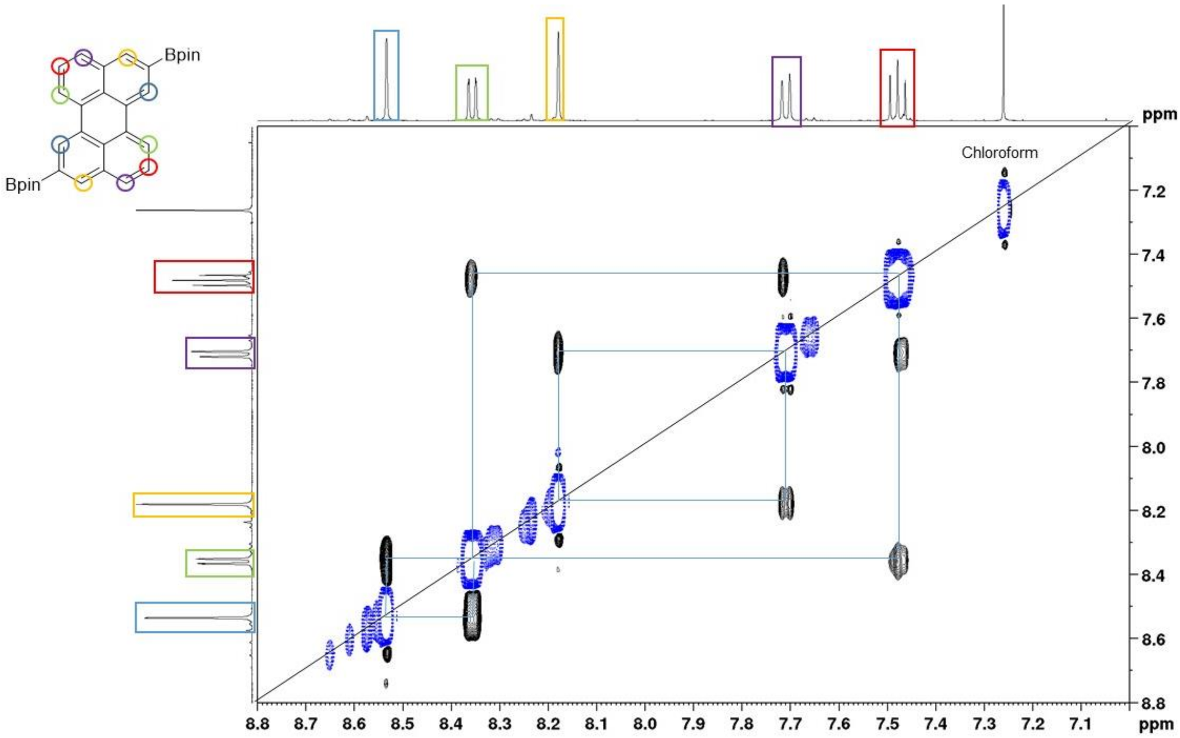

Figure S10: NOESY (500 MHz, 298 K) spectrum **(Bpin)$_2$-Per** recorded in $CDCl_3$.

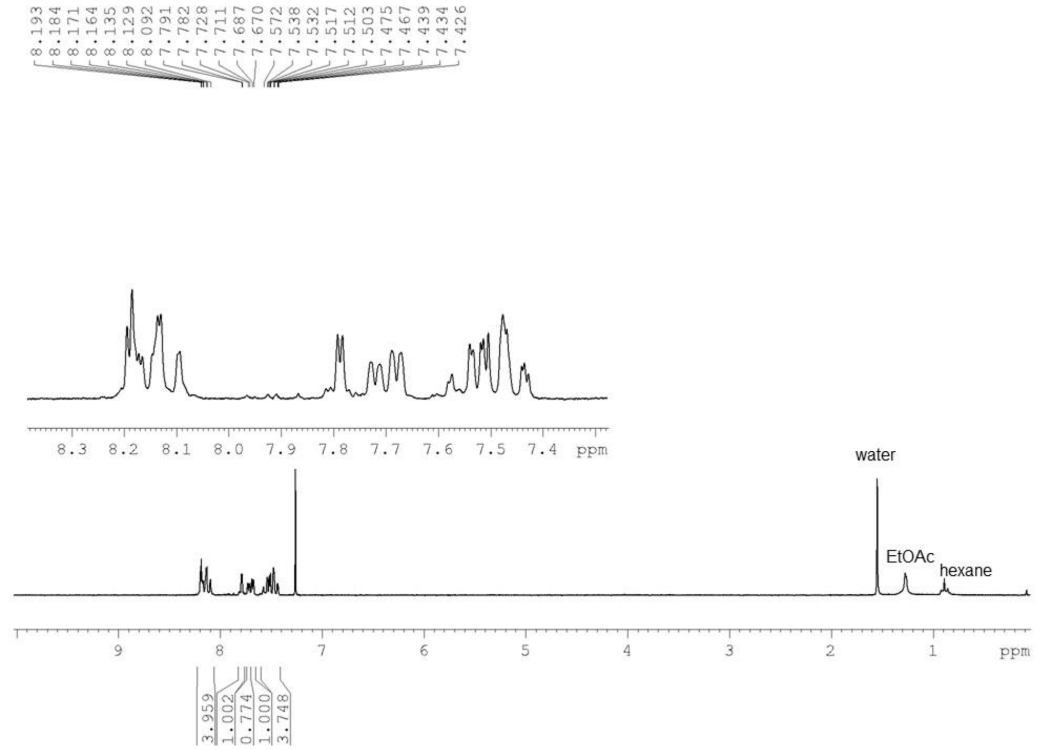

Figure S11: ^{1}H NMR (200 MHz, 298 K) spectrum of **Br-Per** recorded in $CDCl_3$.

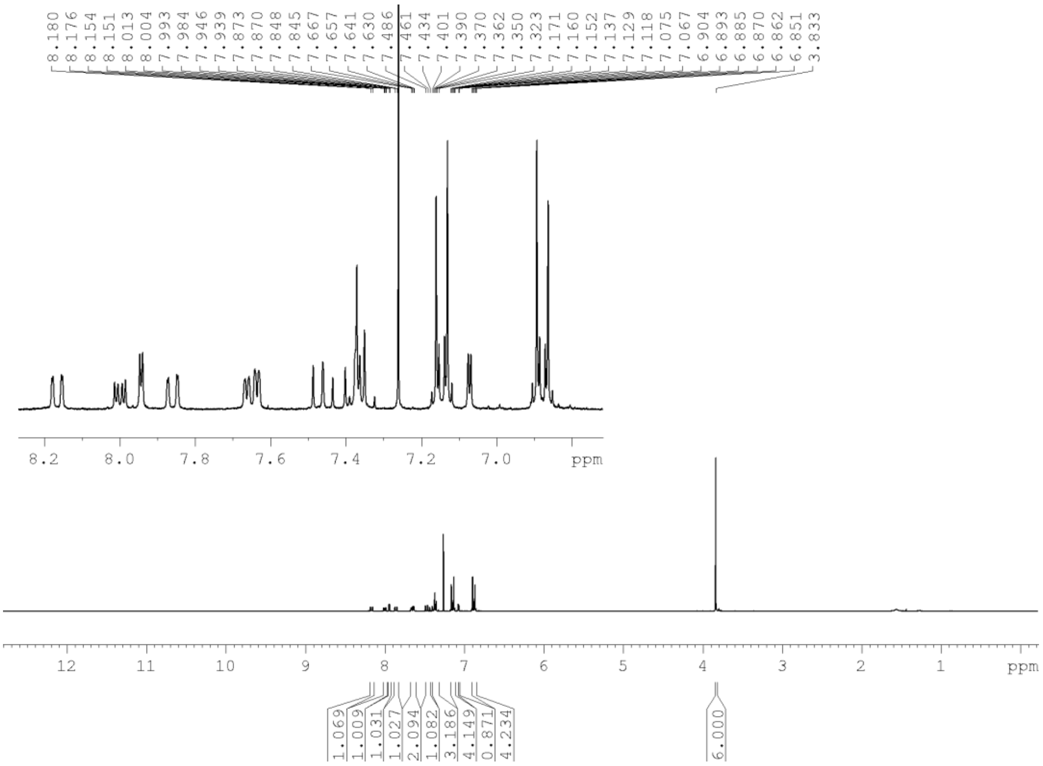

Figure S12: ^{1}H NMR (300 MHz, 298 K) spectrum of **(DPA)-Per** recorded in $CDCl_3$.

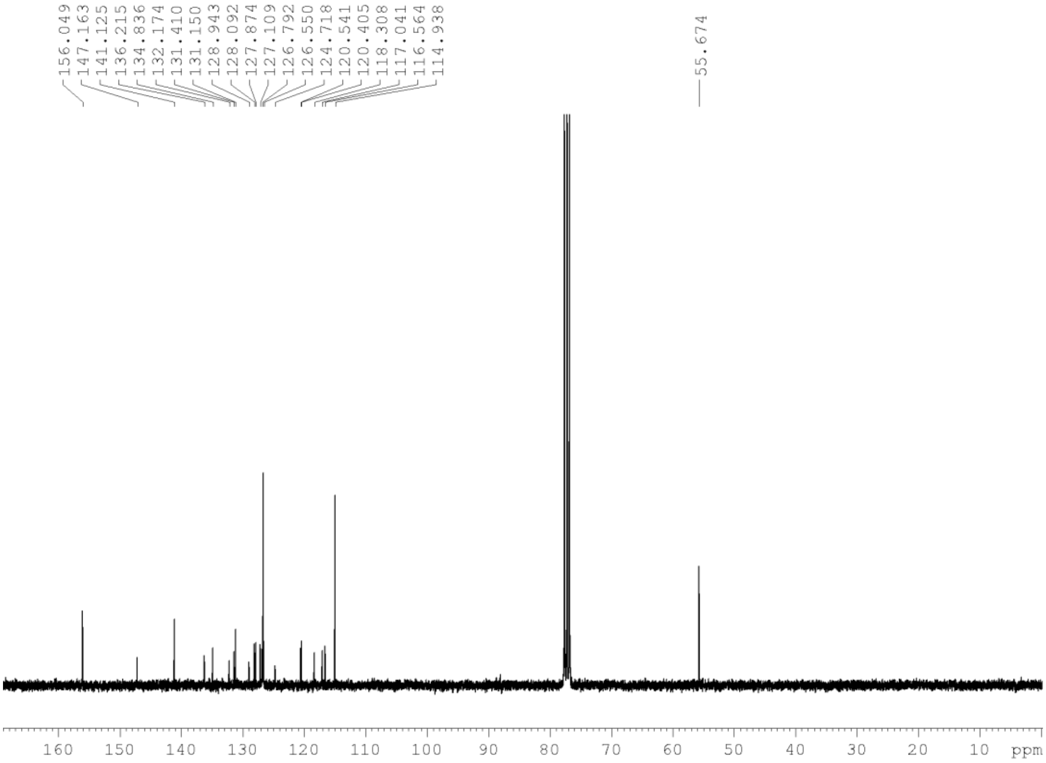

Figure S13: $^{13}C\{^{1}H\}$ NMR (75 MHz, 298 K) spectrum of **(DPA)-Per** recorded in $CDCl_3$.

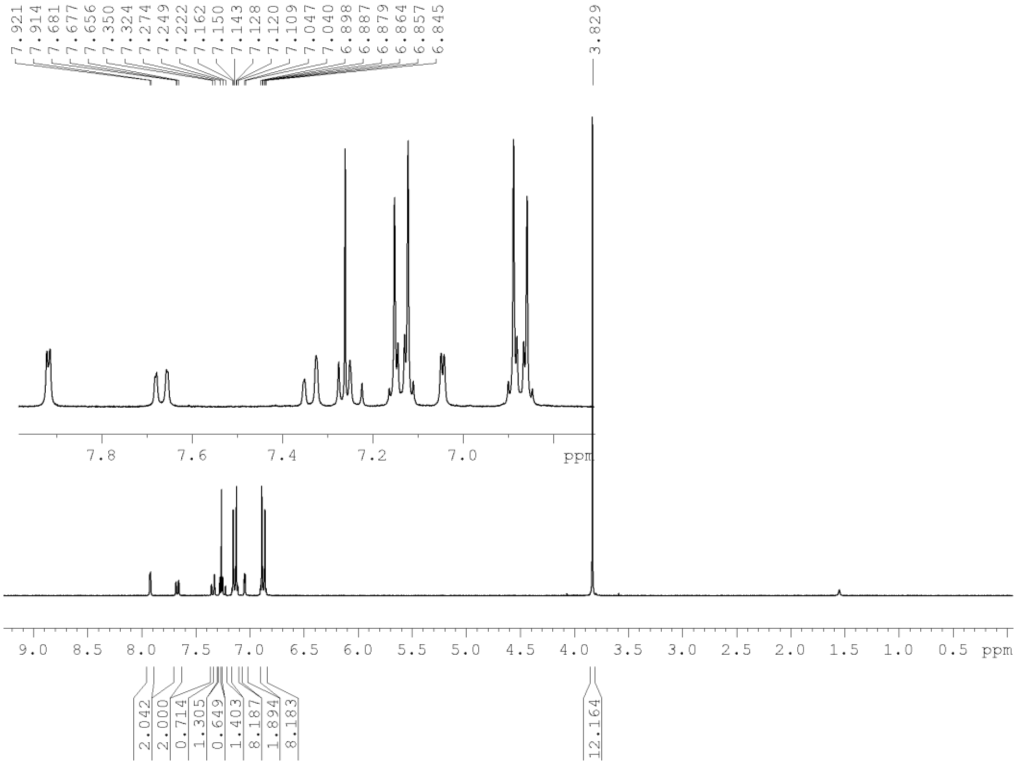

Figure S14: ^{1}H NMR (300 MHz, 298 K) spectrum of **$(DPA)_2$-Per** recorded in $CDCl_3$.

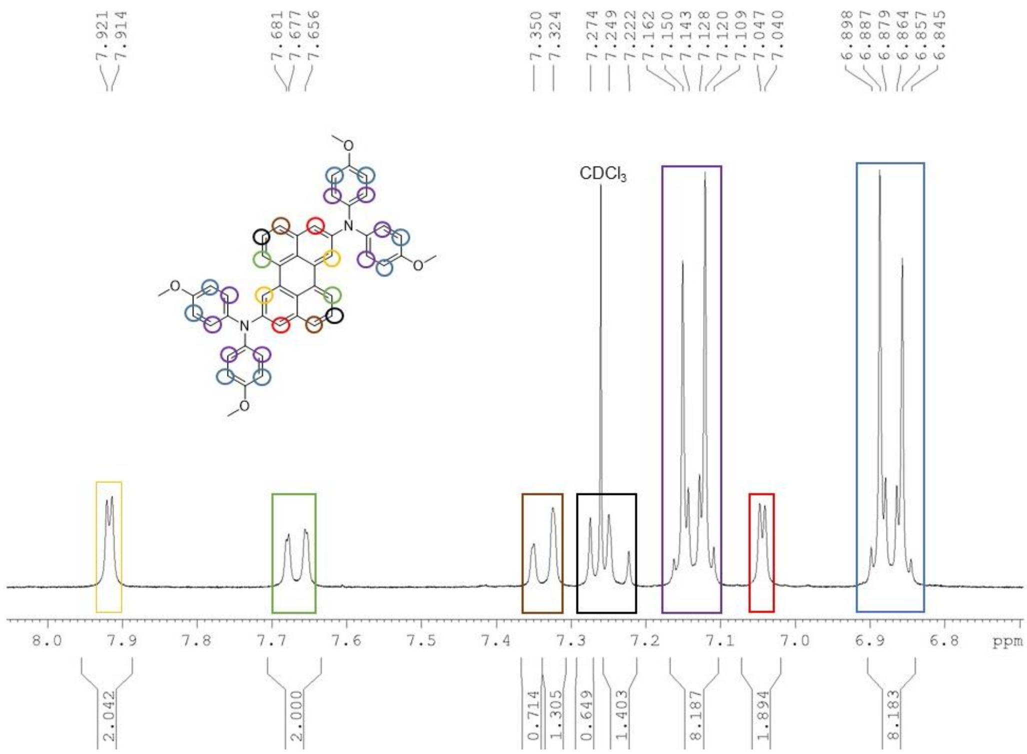

Figure S15: ^{1}H-NMR (300 MHz, 298 K) spectrum of **$(DPA)_2$-Per** in $CDCl_3$.

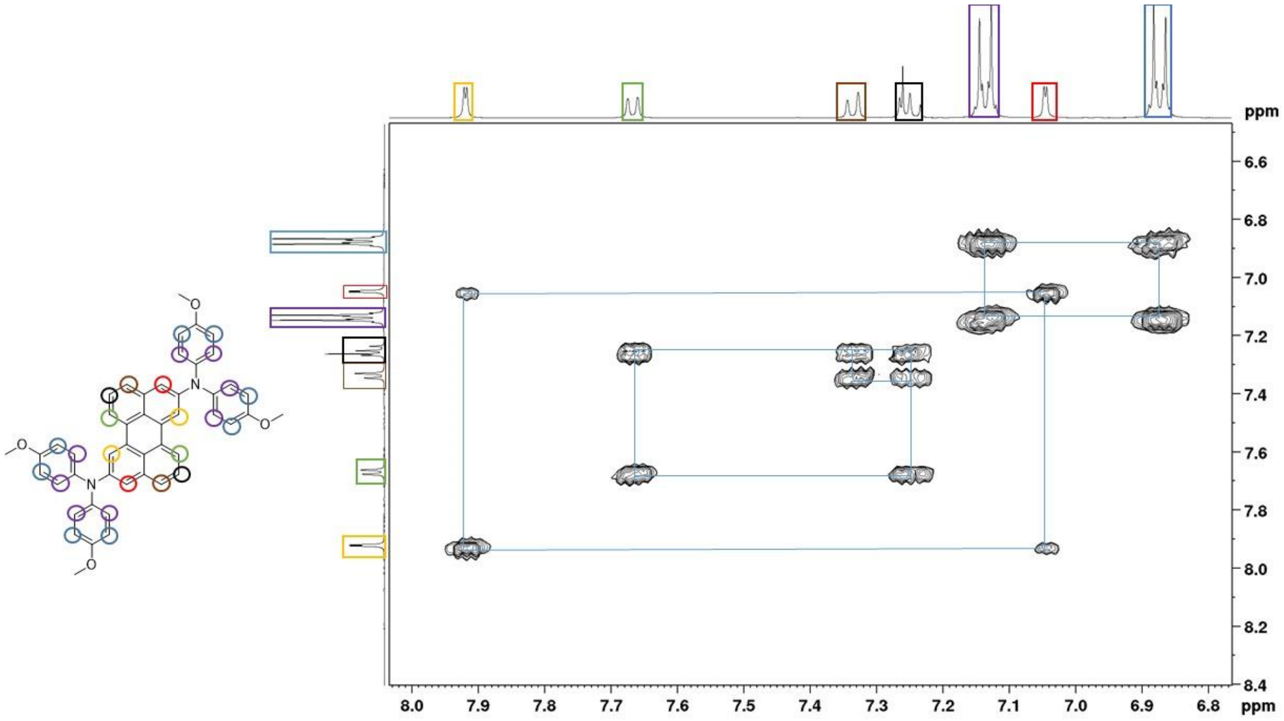

Figure S16: COSY (300 MHz, 298 K) spectrum of **$(DPA)_2$-Per** in $CDCl_3$.

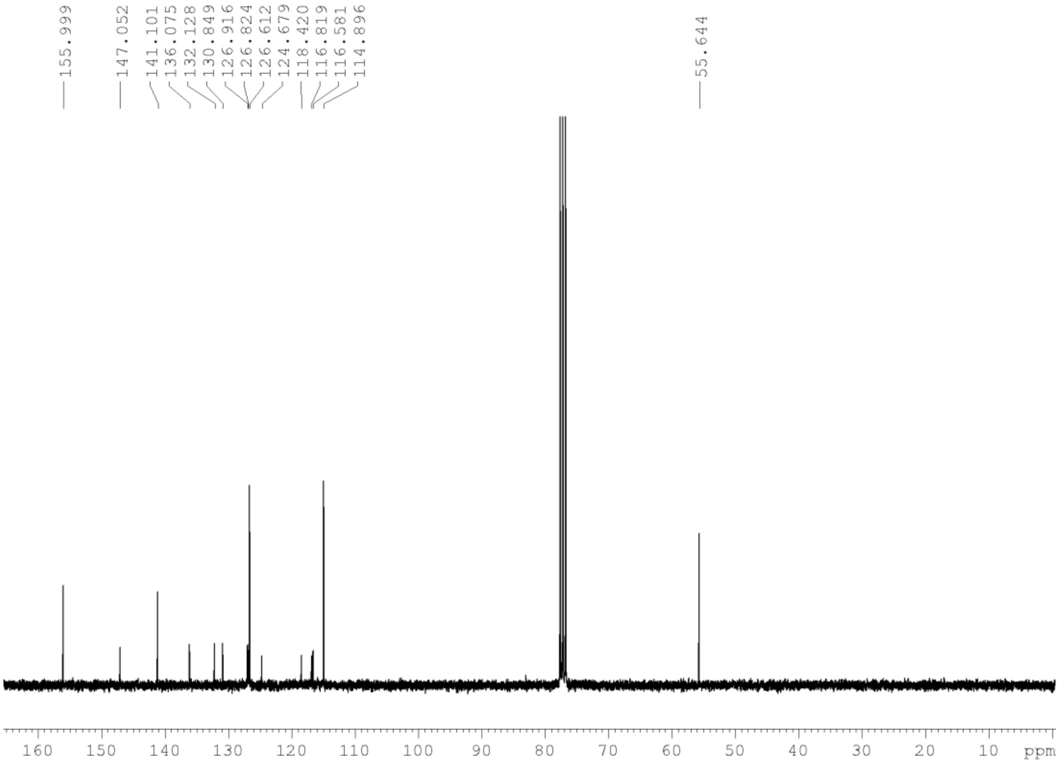

Figure S17: $^{13}C\{^1H\}$ NMR (75 MHz, 298 K) spectrum of **(DPA)$_2$-Per** recorded in $CDCl_3$.

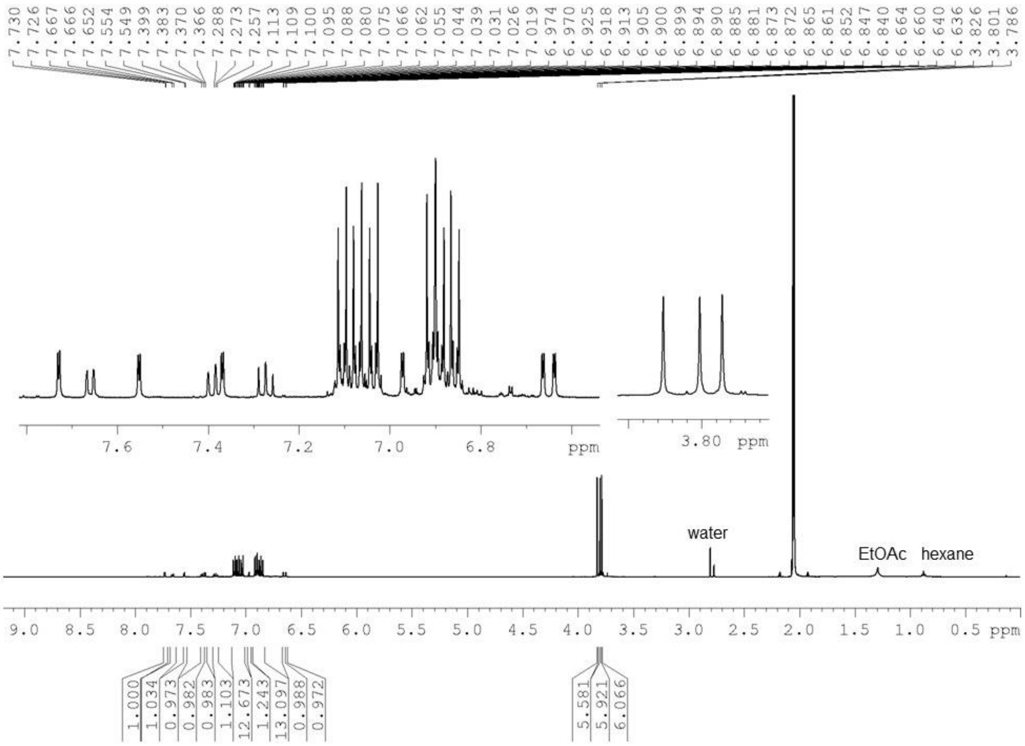

Figure S18: ^1H NMR (500 MHz, 298 K) spectrum of **(DPA)$_3$-Per** recorded in acetone-d_6.

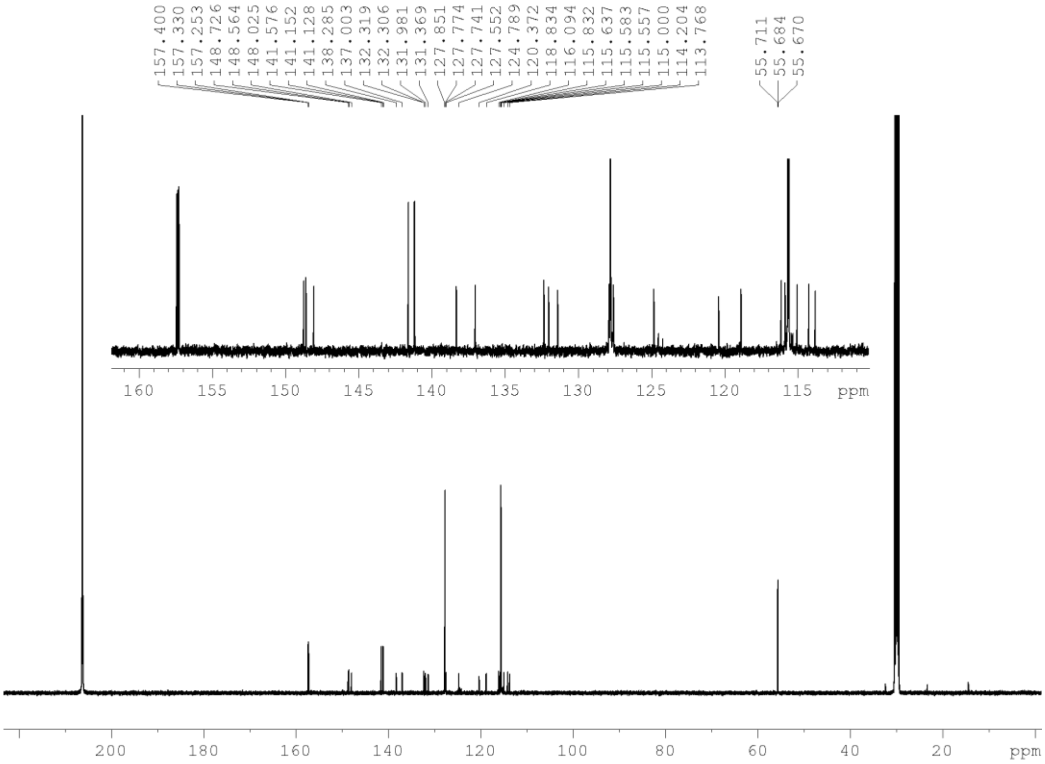

Figure S19: $^{13}C\{^1H\}$ NMR (125 MHz, 298 K) spectrum of **(DPA)$_3$-Per** recorded in acetone-d_6.

Cartesian Coordinates

Compound (DPA)-Per (DFT B3LYP/6-31+G(d,p), S_0)

Total energy: -899568 kcal mol^{-1}

Dipole moment: 2.73 D

C	2.551393	2.846427	-0.042622	C	4.886315	2.319341	-1.864940
C	2.493348	1.433475	0.053785	C	7.318698	2.353283	-0.510174
C	1.297623	0.732137	0.010084	C	6.008982	1.739530	-2.445795
C	0.067714	1.459349	-0.092548	C	7.235277	1.758301	-1.773085
C	0.121835	2.889465	-0.179829	H	8.267057	2.369135	0.023048
C	1.369144	3.556686	-0.167555	H	5.955804	1.282940	-3.428459
C	-1.198973	0.795677	-0.102127	C	3.917333	4.804766	0.567967
C	-2.352794	1.565593	-0.220776	C	4.569102	5.828710	-0.130485
C	-2.474898	-2.783629	0.157750	C	3.391491	5.088092	1.839317
C	-1.088005	3.622315	-0.296632	C	4.703184	7.098844	0.428315
C	1.257367	-0.742966	0.083311	C	3.501932	6.358914	2.390687
C	-0.012206	-1.407731	0.100284	C	4.164870	7.372448	1.688837
C	-1.241582	-0.675160	0.019092	H	5.216700	7.881279	-0.126684
C	-0.053104	-2.839149	0.195187	H	3.094553	6.579695	3.371606
C	-1.305306	-3.504098	0.223627	H	9.085024	1.264577	-1.827140
C	-2.297655	2.967196	-0.320294	H	4.727323	9.203075	1.697525
C	-2.439648	-1.381299	0.054893	H	-3.222131	3.529915	-0.412173
C	2.413781	-1.514820	0.131752	H	-3.327015	1.091878	-0.239928
C	2.368473	-2.917781	0.219086	H	3.297805	-3.478399	0.256751
C	1.159231	-3.572001	0.256230	H	-1.321738	-4.587889	0.297986
H	3.430434	0.906711	0.174497	H	-3.434699	-3.291210	0.182153
H	1.387076	4.637895	-0.250311	N	3.805844	3.496825	0.010583
H	-1.040423	4.705355	-0.366900	O	4.255024	8.600029	2.286189
H	-3.384022	-0.852458	0.004355	O	8.309449	1.185038	-2.397555
H	3.387243	-1.040190	0.096467	H	2.887935	4.302125	2.392443
H	1.113827	-4.655225	0.327069	H	4.979643	5.626086	-1.114078
C	4.954323	2.906058	-0.590358	H	6.254725	3.358681	1.064391
C	6.185808	2.910444	0.078781	H	3.942116	2.315960	-2.399394

Compound (DPA)$_2$-Per (DFT B3LYP/6-31+G(d,p), S_0)

Total energy: -1317582 kcal mol^{-1}

Dipole moment: 0.03 D

C	2.570820	2.785108	-0.244634	H	5.252547	7.779197	-0.840200
C	2.482741	1.420917	0.130197	H	2.475493	7.144687	2.372326
C	1.311051	0.689326	0.007873	H	9.310826	1.077182	-0.411319
C	0.129210	1.342927	-0.470690	H	4.430072	9.425465	0.547517
C	0.213681	2.723779	-0.847211	H	-3.010669	3.190719	-1.863929
C	1.442110	3.417261	-0.738232	H	-3.170792	0.839130	-1.205361
C	-1.119862	0.654567	-0.577834	H	-1.381576	-4.518663	0.922219
C	-2.217921	1.341389	-1.087792	N	3.798063	3.471691	-0.090365
C	-2.490194	-2.814906	0.232456	O	3.845938	8.939723	1.144240
C	-0.942554	3.376560	-1.348613	O	8.656773	0.859093	-1.087959
C	1.244928	-0.741131	0.371127	H	2.441051	4.711728	1.855068
C	-0.013005	-1.418202	0.302681	H	5.190076	5.365595	-1.375854
C	-1.201826	-0.755721	-0.145505	H	6.003797	3.629859	1.405314
C	-0.100349	-2.795325	0.691945	H	4.376381	1.827009	-2.135046
C	-1.341000	-3.473231	0.636380	N	-3.747253	-3.463474	0.208918
C	-2.129473	2.691439	-1.471529	C	-4.695210	-3.168367	-0.812234
C	-2.400854	-1.452440	-0.148291	C	-4.040679	-4.501310	1.141498
C	2.366296	-1.457628	0.779429	C	-6.050260	-3.001699	-0.495005
C	2.281891	-2.812806	1.145556	C	-4.302227	-3.049137	-2.155676
C	1.074449	-3.471791	1.112208	C	-4.555774	-5.729778	0.709190
H	3.374043	0.958048	0.532261	C	-3.836391	-4.305992	2.517546
H	1.486764	4.457516	-1.041796	C	-6.987335	-2.732905	-1.490165
H	-0.871231	4.421698	-1.636534	H	-6.369945	-3.089021	0.538075
H	-3.317619	-0.957202	-0.439169	C	-5.230495	-2.755652	-3.148747
H	3.337614	-0.978855	0.816697	H	-3.258208	-3.183597	-2.418470
H	1.002819	-4.516535	1.401479	C	-4.869752	-6.732844	1.625096
C	5.034537	2.807458	-0.332102	H	-4.718012	-5.897020	-0.350405
C	6.118144	2.987506	0.538487	C	-4.125616	-5.311343	3.432301
C	5.203227	1.969977	-1.447300	H	-3.441618	-3.357263	2.865787
C	7.337302	2.357551	0.298619	C	-6.581792	-2.601336	-2.822079
C	6.410623	1.319069	-1.676435	H	-8.035512	-2.610130	-1.225103
C	7.488483	1.514003	-0.806522	H	-4.926352	-2.659474	-4.185659
H	8.168343	2.512089	0.983814	C	-4.650409	-6.531480	2.990618
H	6.539618	0.669002	-2.535337	H	-5.273411	-7.679435	1.271538
C	3.811900	4.867426	0.200536	H	-3.966421	-5.162298	4.495023
C	4.597604	5.749322	-0.551982	O	-7.453046	-2.326729	-3.841162
C	3.050848	5.385047	1.261777	O	-4.928914	-7.479779	3.937082
C	4.632803	7.109345	-0.247582	H	-8.344459	-2.241325	-3.478842
C	3.064311	6.743543	1.554212	H	-5.277069	-8.267870	3.500118
C	3.861616	7.614662	0.802783	H	3.181128	-3.335058	1.459224

Compound (DPA)$_3$-Per (DFT B3LYP/6-31+G(d,p), S_0)

Total energy: -1735596 kcal mol^{-1}

Dipole moment: 2.31 D

C	2.571883	2.844982	-0.056914	H	4.498698	9.170307	2.033774
C	2.523337	1.430633	0.023300	H	-3.158541	3.477988	-0.888412
C	1.341776	0.718301	-0.117689	H	-3.251324	1.036174	-0.747918
C	0.114986	1.435013	-0.308542	H	-1.240928	-4.618719	-0.090376
C	0.160556	2.866224	-0.381517	N	3.812069	3.509694	0.098757
C	1.396510	3.545025	-0.266824	O	4.002621	8.538183	2.569992
C	-1.141935	0.761408	-0.422400	O	8.471427	1.416177	-2.211572
C	-2.288590	1.520600	-0.636369	H	2.728266	4.211834	2.439471
C	-2.416330	-2.832159	-0.272778	H	5.012381	5.701772	-0.874994
C	-1.042751	3.589210	-0.590558	H	6.225823	3.418887	1.234701
C	1.308171	-0.758867	-0.056475	H	4.067682	2.392969	-2.332653
C	0.046385	-1.426568	-0.142668	N	3.649121	-3.647297	0.273434
C	-1.180177	-0.711042	-0.310619	C	3.779586	-4.938727	-0.315742
C	0.009345	-2.856486	-0.060837	C	4.700055	-3.155003	1.097281
C	-1.230696	-3.535427	-0.140207	C	4.251987	-6.026298	0.429667
C	-2.239630	2.923495	-0.721310	C	3.449601	-5.143417	-1.665795
C	-2.373528	-1.418013	-0.351337	C	6.033645	-3.244532	0.674425
C	2.465040	-1.515887	0.066256	C	4.433160	-2.583820	2.352831
C	2.433851	-2.929883	0.152382	C	4.403017	-7.281376	-0.158313
C	1.213992	-3.582848	0.099179	H	4.509012	-5.885914	1.474270
H	3.454093	0.912534	0.213976	C	3.575998	-6.397942	-2.249997
H	1.410745	4.627156	-0.339313	H	3.085988	-4.307941	-2.254797
H	-0.999554	4.673224	-0.648453	C	7.070424	-2.789418	1.485671
H	-3.316590	-0.895014	-0.435892	H	6.257437	-3.678806	-0.294158
H	3.435254	-1.037320	0.086460	C	5.463850	-2.104676	3.154323
H	1.170566	-4.663966	0.173604	H	3.407206	-2.514214	2.698419
C	4.997679	2.970258	-0.475846	C	4.059942	-7.475385	-1.499103
C	6.205302	2.997178	0.235395	H	4.775398	-8.114020	0.435080
C	4.992555	2.414340	-1.765969	H	3.319548	-6.557318	-3.292012
C	7.375636	2.495675	-0.329198	C	6.791255	-2.211394	2.727831
C	6.153711	1.889046	-2.322843	H	8.099503	-2.870955	1.141345
C	7.355484	1.933294	-1.609297	H	5.256815	-1.661081	4.122498
H	8.304242	2.528492	0.237152	O	4.176762	-8.684726	-2.129756
H	6.148723	1.456808	-3.317879	O	7.767585	-1.737553	3.563620
C	3.862350	4.799048	0.704900	H	4.512491	-9.335964	-1.500182
C	4.534664	5.860428	0.086038	H	8.629268	-1.884954	3.152818
C	3.248705	5.027102	1.947864	N	-3.667281	-3.493705	-0.315554
C	4.603571	7.112433	0.695634	C	-4.709823	-3.014433	-1.158480
C	3.294414	6.279500	2.548199	C	-3.864596	-4.709416	0.401834
C	3.978644	7.330432	1.926511	C	-6.030184	-2.955954	-0.691508
H	5.134089	7.924347	0.202273	C	-4.446471	-2.600540	-2.474952
H	2.819301	6.456909	3.507229	C	-4.419799	-5.831547	-0.225999
H	9.224880	1.520035	-1.616148	C	-3.522247	-4.802107	1.761170

C	-7.058881	-2.506668	-1.516613	H	-8.078222	-2.471638	-1.137399
H	-6.250477	-3.270030	0.323330	H	-5.262094	-1.804196	-4.308696
C	-5.466405	-2.127591	-3.293485	C	-4.282651	-7.094593	1.833729
H	-3.430889	-2.647087	-2.853670	H	-5.075010	-7.871104	-0.018334
C	-4.638801	-7.010600	0.484955	H	-3.450746	-6.055754	3.517242
H	-4.687799	-5.777347	-1.275871	O	-7.747763	-1.621523	-3.673385
C	-3.716808	-5.982663	2.468030	O	-4.463540	-8.226285	2.582248
H	-3.094541	-3.938879	2.260195	H	-8.601209	-1.639595	-3.221217
C	-6.781873	-2.083266	-2.820278	H	-4.852161	-8.912779	2.024665
